# PTPN23 ubiquitination by WDR4 suppresses EGFR and c-MET degradation to define a lung cancer therapeutic target

**DOI:** 10.1038/s41419-023-06201-4

**Published:** 2023-10-11

**Authors:** Shaifali Singh, Nai Yang Yeat, Ya-Ting Wang, Shu-Yu Lin, I-Ying Kuo, Kuen-Phon Wu, Won-Jing Wang, Wen-Ching Wang, Wu-Chou Su, Yi-Ching Wang, Ruey-Hwa Chen

**Affiliations:** 1 Institute of Biological Chemistry, Academia Sinica, Taipei, 115 Taiwan; 2 Chemical Biology and Molecular Biophysics Program, Taiwan International Graduate Program, Academia Sinica, Taipei, 115 Taiwan; 3 Institute of Molecular & Cellular Biology and Department of Life Science, National Tsing Hua University, Hsinchu, 300 Taiwan; 4 Department of Chemistry, National Tsing Hua University, Hsinchu, 300 Taiwan; 5 Department of Pharmacology, College of Medicine, National Cheng Kung University, Tainan, 701 Taiwan; 6 Institute of Basic Medical Sciences, College of Medicine, National Cheng Kung University, Tainan, 701 Taiwan; 7 Institute of Biochemistry and Molecular Biology, National Yang Ming Chiao Tung University, Taipei, 112 Taiwan; 8 Division of Oncology, Department of Internal Medicine, National Cheng Kung University Hospital, College of Medicine, National Cheng Kung University, Tainan, 701 Taiwan

**Keywords:** Cancer, Cancer therapy, Ubiquitylation

## Abstract

Aberrant overexpression or activation of EGFR drives the development of non-small cell lung cancer (NSCLC) and acquired resistance to EGFR tyrosine kinase inhibitors (TKIs) by secondary EGFR mutations or c-MET amplification/activation remains as a major hurdle for NSCLC treatment. We previously identified WDR4 as a substrate adaptor of Cullin 4 ubiquitin ligase and an association of WDR4 high expression with poor prognosis of lung cancer. Here, using an unbiased ubiquitylome analysis, we uncover PTPN23, a component of the ESCRT complex, as a substrate of WDR4-based ubiquitin ligase. WDR4-mediated PTPN23 ubiquitination leads to its proteasomal degradation, thereby suppressing lysosome trafficking and degradation of wild type EGFR, EGFR mutant, and c-MET. Through this mechanism, WDR4 sustains EGFR and c-MET signaling to promote NSCLC proliferation, migration, invasion, stemness, and metastasis. Clinically, PTPN23 is downregulated in lung cancer and its low expression correlates with WDR4 high expression and poor prognosis. Targeting WDR4-mediated PTPN23 ubiquitination by a peptide that competes with PTPN23 for binding WDR4 promotes EGFR and c-MET degradation to block the growth and progression of EGFR TKI-resistant NSCLC. These findings identify a central role of WDR4/PTPN23 axis in EGFR and c-MET trafficking and a potential therapeutic target for treating EGFR TKI-resistant NSCLC.

## Introduction

Non-small cell lung cancer (NSCLC) is the leading cause of death among cancer-related mortality worldwide and comprises ~85% of lung cancer cases [1, 2]. Overexpression or activating mutation of EGFR, such as L858R point mutation or exon 19 deletion, is a major driving force for NSCLC development [3]. Although patients harboring these mutations respond initially to 1st-generation EGFR tyrosine kinase inhibitors (TKIs), such as gefitinib and erlotinib, most of them develop acquired resistance with variable periods [4–8]. Since EGFR T790M secondary mutation is the most common mechanism of acquired resistance [9, 10], the 3rd-generation EGFR TKIs, such as osimertinib (AZD9291) and rociletinib, are developed to overcome the T790M-mediated TKI resistance [11, 12]. However, the vast majority of patients ultimately develop new acquired resistance to 3rd-generation EGFR TKIs [13, 14]. Furthermore, resistance to 1st- or 3rd-generation EGFR TKIs can be mediated by various EGFR-independent mechanisms, thus complicating the selection of effective agents for further treatment. Among these mechanisms, activation of the bypass pathways, such as amplification of c-MET, represents a major category [13, 15–17]. The diverse mechanisms of EGFR TKI resistance highlight an unmet need for developing a novel type of therapeutic strategies to improve the clinical outcomes of NSCLC.

Recently, targeted protein degradation has emerged as a new and promising approach for developing targeted therapy drugs. This strategy allows target removal from tumor cells and offers several advantages over the traditional, occupancy-driven pharmacology [18]. Protein degradation is achieved by introducing a chimeric molecule that targets the protein of interest (POI) to undergo proteasome-, lysosome-, or autophagy-mediated degradation [19]. In addition, re-activating cell-intrinsic degradation machinery is an alternative approach for POI degradation. The cellular degradation mechanism for EGFR is primarily mediated by endosome-lysosome pathway [20]. Following ligand binding, the activated EGFR is endocytosed and transported to the early endosome. While a portion of EGFR is recycled at this point, a great fraction is sorted to multivesicular body (MVB)/late endosome, which then fuses with lysosome to facilitate EGFR degradation. Since the internalized EGFR remains competent for signaling [21, 22], trafficking to late endosome/lysosome represents a determining step for signal termination. Furthermore, EGFR mutants display specific differences in endocytosis from their wild type counterpart by increasing the recycling pool and decreasing the degradation pool, thus resulting in an enhanced signaling output [23, 24]. Thus, diverting the endocytic route from recycling to degradation would be a strategy for targeting the EGFR mutants that cause resistance to EGFR TKIs.

The ESCRT pathway plays a key role in determining the lysosomal degradation fate of EGFR and many other cell surface receptors. ESCRTs comprise a series of multi-protein complexes (ESCRT-0, -I, -II, -III and VPS4 complex), which act consecutively and coordinately to facilitate cargo binding as well as inward budding and scission of the endosome membrane, thereby facilitating cargo sorting and MVB formation [25, 26]. PTPN23, also known as HD-PTP (His domain protein tyrosine phosphatase), is critical for trafficking of EGFR to MVB and lysosome [27]. This multi-domain protein functions as a scaffold for recruiting several ESCRT components, including ESCRT-0 subunit STAM2, ESCRT-I subunits TSG101 and UBAP1, and ESCRT-III subunit CHMP4B [28–31]. Intriguingly, CHMP4B and STAM2 bind PTPN23 Bro1 domain competitively, suggesting the dual roles of PTPN23 in ESCRTs assembly and cargo transport from early- to late-acting ESCRTs [28, 31]. In addition, a recent study revealed a function of PTPN23 in promoting endosome maturation through Rab5 inactivation [32], which also contributes to the direction of EGFR towards a lysosomal degradation fate. Besides EGFR, PTPN23 promotes MVB sorting and lysosomal degradation of other oncogenic receptors, such as PDGFR and integrins [33, 34], and elicits tumor suppressive activities by regulating cell adhesion, migration, and invasion [33, 35, 36]. Accordingly, mice with hemizygous deletion of *Ptpn23* are prone to develop spontaneous lung adenoma and B-cell lymphoma and promote the onset and progression of Myc-driven lymphoma. Furthermore, downregulation of PTPN23 protein is frequently observed in various human cancers, including lung cancer [37]. However, the mechanism that regulates PTPN23 expression in cancer has not been fully understood.

Our previous study identified WDR4 as a substrate adaptor of Cullin 4 (Cul4) ubiquitin ligase. WDR4 is highly expressed in lung cancer and promotes lung cancer progression and metastasis by targeting PML tumor suppressor for ubiquitination [38]. In this study, we identify PTPN23 as a novel substrate of WDR4-based Cul4 ligase and WDR4-mediated PTPN23 proteolysis prevents the lysosomal degradation of not only wild type EGFR but also TKI-resistant EGFR mutant and c-MET, thereby sustaining the downstream signaling of these RTKs to support various tumor-promoting functions. In human lung cancers, WDR4/PTPN23 axis is upregulated in lung cancer and associated with adverse prognosis. Furthermore, we design a PTPN23 peptide that competes with endogenous PTPN23 for binding WDR4 and demonstrate its efficacies in stabilizing PTPN23, promoting the degradation of EGFR, EGFR mutant, and c-MET, and suppressing the growth and progression of EGFR TKI-resistant NSCLC. This study identifies a key role of WDR4/PTPN23 axis in NSCLC progression by suppressing lysosomal degradation of EGFR and c-MET and suggests a previously unappreciated strategy for treating EGFR TKI-resistant NSCLC by reactivating the degradation of EGFR mutant and c-MET.

## Results

### Identification of PTPN23 as a substrate of WDR4-based Cul4 ubiquitin ligase

**Fig. 1:**
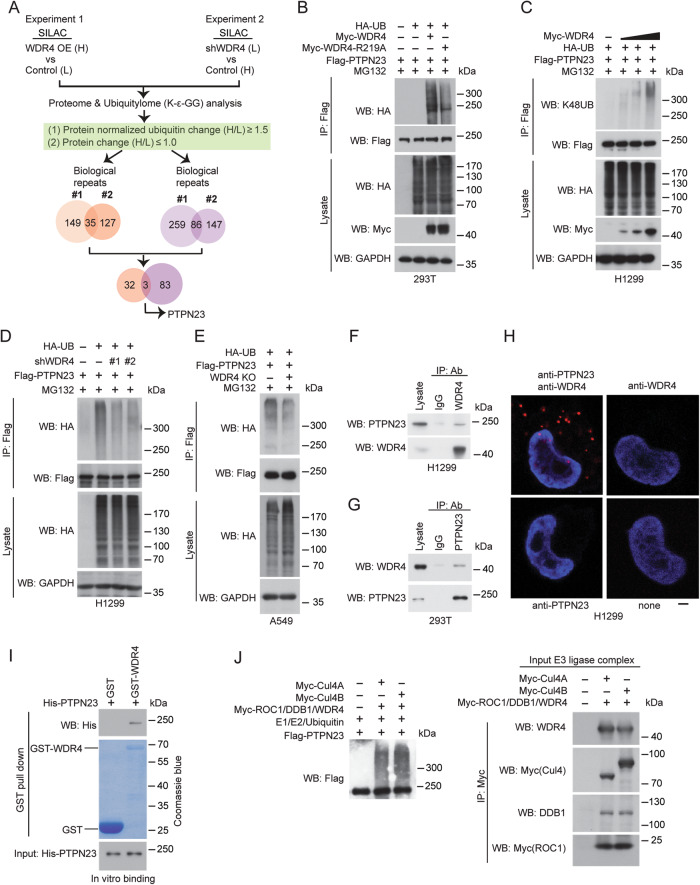
Identification of PTPN23 as a substrate of WDR4-based E3 ligase. **A** Schematic presentation of the design of quantitative proteome and ubiquitylome analyses on control and WDR4 overexpressing (OE) A549 cells or control and WDR4 knockdown A549 cells. The criteria for hit selection and the number of hits recovered are shown. **B**, **C** Immunoprecipitation analysis for PTPN23 ubiquitination in HEK293T (**B**) and H1299 (**C**) cells transfected with the indicated constructs. **D**, **E** Immunoprecipitation analysis of PTPN23 ubiquitination in H1299 cells stably expressing control or WDR4 shRNAs (**D**) or A549 control or WDR4 KO cells (**E**) and transiently transfected with the indicated constructs. The efficient knockdown and knockout of WDR4 in these cells are shown in Figs. 2B and C, respectively. **F**, **G** Immunoprecipitation analysis of the interaction between endogenous PTPN23 and endogenous WDR4 in indicated cells. **H** Representative PLA images for the interaction between endogenous WDR4 and endogenous PTPN23 in H1299 cells. Antibodies used are indicated. Bar, 10 μm. **I** GST pull down analysis for the in vitro interaction between GST-WDR4 and His-PTPN23. **J** In vitro ubiquitination assay for PTPN23. His-PTPN23 purified from baculovirus was incubated with E1, E2, ubiquitin and/or WDR4-based Cul4A or Cul4B complex purified from transfected HEK293T cells. The integrity of input E3 ligase complex is shown on the right.

To enhance our understanding of the mechanistic basis of tumor-promoting functions of WDR4-based Cul4 ubiquitin ligase, we aimed to identify its substrates in lung cancer cells using an unbiased approach. To this end, a previously established protocol [39] was employed to access ubiquitylome changes caused by WDR4 overexpression and knockdown in A549 NSCLC cells. As shown in Fig. 1A, SILAC-labeled WDR4 overexpressing or knockdown cells along with their control counterparts were subjected to liquid chromatography-tandem mass spectrometry (LC-MS/MS) for proteome analysis. For ubiquitylome analysis, trypsin digested peptides were immunoprecipitated with a K-ε-GG antibody, which recognizes the Gly-Gly remnant of ubiquitinated Lys after trypsin digestion, and then subjected to LC-MS/MS. Proteins displayed [protein-normalized K-ε-GG peptide changes] (H/L) ≥ 1.5 and [protein changes] ≤1.0 were selected as hits. We performed two biological repeats for each set of experiments and PTPN23 was recovered from all four datasets (Fig. 1A and Supplementary Table S1). In the validation experiment, we showed that WDR4 overexpression in HEK293T cells increased PTPN23 ubiquitination, whereas WDR4 R219A mutant, which has a reduced ability to form Cul4 E3 ligase complex [38], showed a decreased ubiquitination effect (Fig. 1B). WDR4 overexpression in an NSCLC cell line H1299 also increased PTPN23 K48-linked ubiquitination in a dose-dependent manner (Fig. 1C). Conversely, WDR4 knockdown in H1299 cells diminished PTPN23 ubiquitination (Fig. 1D). To further validate the critical role of WDR4 in PTPN23 ubiquitination, we established WDR4 knockout (KO) A549 cells by CRISPR technology and found a substantial reduction in PTPN23 ubiquitination by WDR4 KO (Fig. 1E). Next, we determined the association of WDR4 with PTPN23. Immunoprecipitation analysis demonstrated the interaction of endogenous WDR4 with endogenous PTPN23 in H1299 and HEK293T cells (Fig. 1F, G). This interaction was further confirmed by proximity ligation assay (PLA) with antibodies to PTPN23 and WDR4 (Fig. 1H). In addition, baculovirally purified PTPN23 was readily pulled down by bacterially purified GST-WDR4 (Fig. 1I). Furthermore, purified Cul4A or Cul4B E3 ligase complex containing WDR4 could support the ubiquitination of PTPN23 in vitro (Fig. 1J). These data collectively identify PTPN23 as a substrate of WDR4-based Cul4 ubiquitin ligase.

### WDR4 promotes PTPN23 proteasomal degradation

**Fig. 2:**
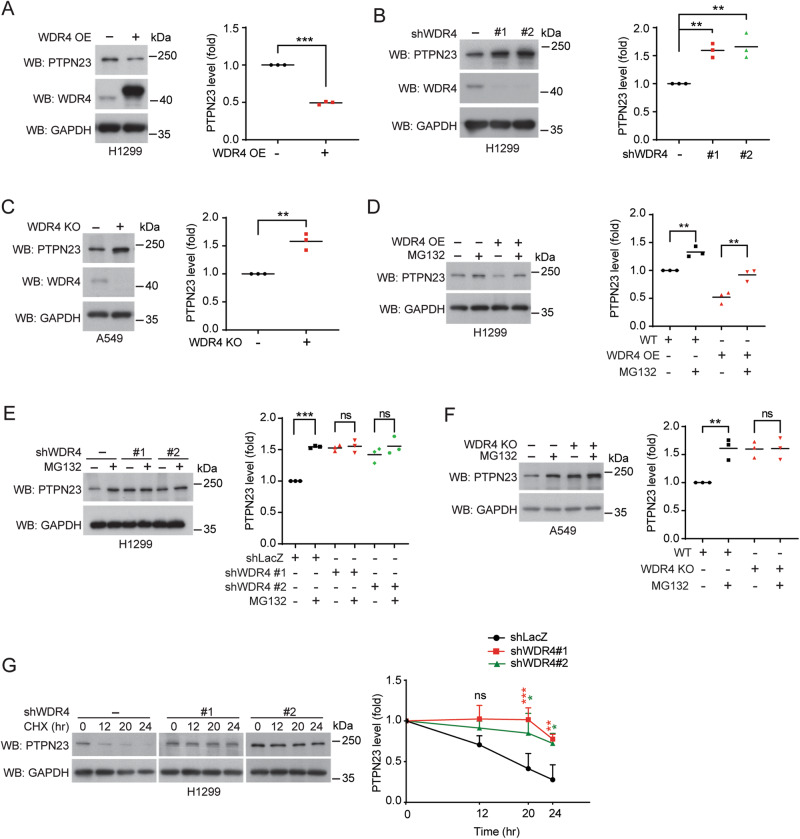
WDR4 promotes PTPN23 proteasomal degradation. **A**–**C** Western blot analysis of PTPN23 levels in H1299 cells overexpressing WDR4 (**A**), H1299 cells stably expressing WDR4 shRNAs (**B**), or WDR4 KO A549 cells (**C**). The blots are representatives of three independent experiments and quantitative data are shown on the right. **D**–**F** Western blot analysis of PTPN23 levels in indicated cells treated with or without 10 μM MG132 for 16 h. The blots are representatives of three independent experiments and quantitative data are shown on the right. **G** Western blot analysis of PTPN23 levels in H1299 cells stably expressing control or WDR4 shRNAs and treated with 20 μg/ml cycloheximide for indicated time periods. Quantitative data are shown on the right. Data in (**A**–**C**) are represented as individual points and mean and data in (**G**) is mean ± SD, *n* = 3. *P*-values are determined by two-sided Student’s *t*-test (**A**, **C**), one way ANOVA with Tukey’s post hoc test (**B**, **D**, **E**, **F**), or two-way ANOVA with Tukey’s post hoc test (**G**), *P < 0.05, **P < 0.01, ***P < 0.001. ns: not significant.

Consistent with the effect of WDR4 on promoting PTPN23 K48-ubiquitination, WDR4 overexpression in H1299 cells decreased the abundance of PTPN23 (Fig. 2A), whereas WDR4 knockdown in this cell line elevated PTPN23 protein levels without affecting its mRNA expression (Fig. 2B and Supplementary Fig. S1A). WDR4 knockdown in another NSCLC cell line H1975 also upregulated PTPN23 expression (Supplementary Fig. S1B). A similar finding was observed in WDR4 KO A549 cells (Fig. 2C). Furthermore, WDR4 overexpression in H1299 cells enhanced PTPN23 proteasomal degradation, whereas WDR4 knockdown in H1299 or knockout in A549 cells showed an opposite effect (Fig. 2D–F). WDR4 knockdown also decreased the turnover of PTPN23 protein (Fig. 2G). Thus, WDR4 promotes the proteasomal degradation of PTPN23.

Besides acting as a substrate adaptor for Cul4 ubiquitin ligase, WDR4 forms a complex with the catalytic subunit of m^7^G methyltransferase METTL1 to assist tRNA methylation at nucleotide 46 [40, 41]. WDR4 R170L mutation was discovered from microcephalic primordial dwarfism patients showing an impairment of tRNA m^7^G methylation [42]. We found that WDR4 R170L promoted PTPN23 degradation as efficiently as the wild type WDR4 (Supplementary Fig. S1C). Furthermore, depletion of METTL1 did not affect PTPN23 abundance (Supplementary Fig. S1D). Thus, WDR4 promotes PTPN23 degradation through an m^7^G methyltransferase-independent mechanism.

### WDR4/PTPN23 axis suppresses EGFR lysosome trafficking and degradation

**Fig. 3:**
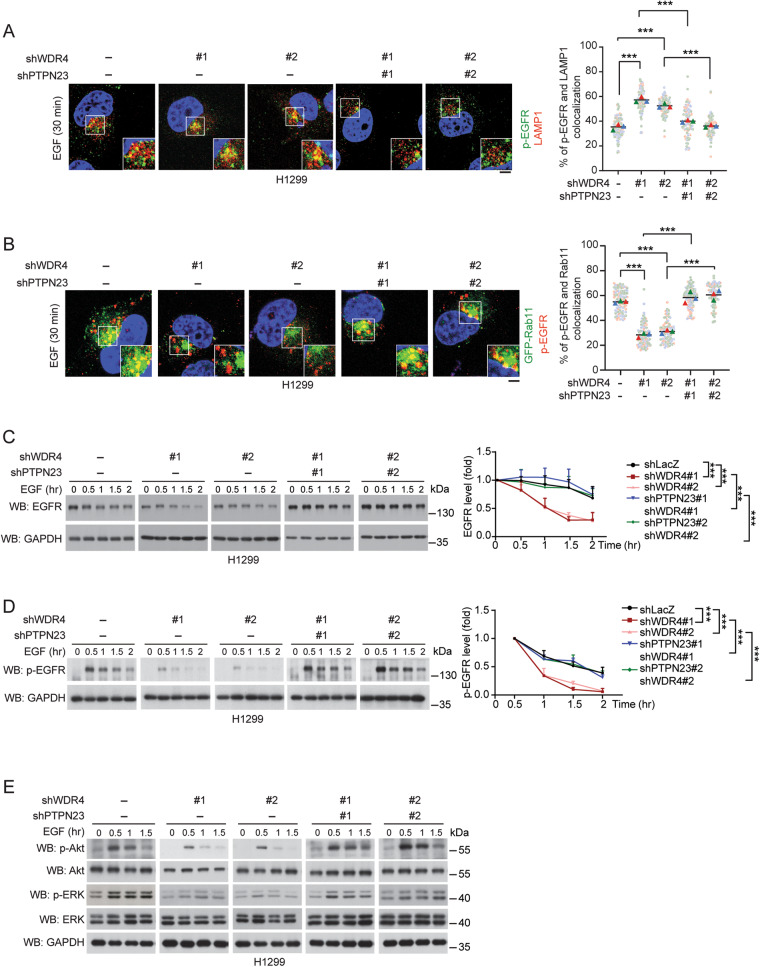
WDR4/PTPN23 axis inhibits EGFR lysosomal degradation to sustain EGFR signaling. **A**, **B** Immunofluorescence staining for the colocalization of p-EGFR with LAMP1 (**A**) or with GFP-Rab11 (**B**) in H1299 cells stably expressing various shRNAs and stimulated with EGF (100 ng/ml) for 30 min. GFP-Rab11 was introduced by transient transfection. The knockdown efficiencies of various shRNAs are shown in Supplementary Fig. S2A. Representative confocal images are shown on the left and quantitative data are on the right. Bar, 20 μm. Data are represented as mean, *n* = 3 (30 cells per group per experiment were counted). *P*-values are determined by two-way ANOVA with Tukey’s post hoc test, ***P < 0.001. **C**, **D** Western blot analysis of EGFR and p-EGFR in H1299 cells stably expressing various shRNAs and stimulated with EGF (100 ng/ml) for indicated time periods. The blots are representatives of three independent experiments and quantitative data are shown on the right. Data are mean ± SD, *n* = 3. *P*-values are determined by two-way ANOVA with Tukey’s post hoc test, ***P < 0.001. **E** Western blot analysis of indicated proteins in H1299 cells stably expressing various shRNAs and stimulated with EGF (100 ng/ml) for indicated time periods.

PTPN23 is critical for lysosome trafficking of ligand-induced EGFR [28, 31]. We thus interrogated the effect of WDR4/PTPN23 axis on EGF-induced p-EGFR trafficking using WDR4 knockdown and WDR4/PTPN23 double knockdown cells (Supplementary Fig. S2A). Notably, WDR4 knockdown in H1299 cells, which express a wild type EGFR, increased EGF-induced colocalization of p-EGFR with lysosome marker LAMP1, and this effect was reversed by WDR4/PTPN23 double knockdown (Fig. 3A). WDR4 knockdown did not affect EGF internalization (Supplementary Fig. S2B). Intriguingly, WDR4 knockdown decreased EGF-induced colocalization of p-EGFR with recycling endosome marker Rab11, suggesting that the blockage of lysosome trafficking by WDR4 deficiency redirected EGFR to recycling endosome. Again, this effect of WDR4 knockdown was rescued by WDR4/PTPN23 double knockdown (Fig. 3B). In line with these findings, WDR4 knockdown increased the EGF-induced turnover of EGFR and p-EGFR and WDR4/PTPN23 double knockdown reversed these effects (Fig. 3C, D). Importantly, blockage of lysosomal degradation using bafilomycin A1 abrogated the suppressive effects of WDR4/PTPN23 axis on EGF-induced EGFR degradation (Supplementary Fig. S2C), confirming the involvement of lysosome. In contrast, the WDR4/PTPN23 axis did not affect *EGFR* mRNA and protein abundance in H1299 cells without EGF treatment (Supplementary Fig. S2D, E). We also showed that WDR4 knockdown diminished EGFR downstream signaling, as monitored by ERK and Akt phosphorylation, which were reverted by WDR4/PTPN23 double knockdown (Fig. 3E). Our findings indicate that WDR4-mediated PTPN23 ubiquitination impairs lysosome trafficking and degradation of wild type EGFR to sustain its downstream signaling.

### WDR4/PTPN23 axis suppresses lysosome trafficking and degradation of EGFR mutant and c-MET

**Fig. 4:**
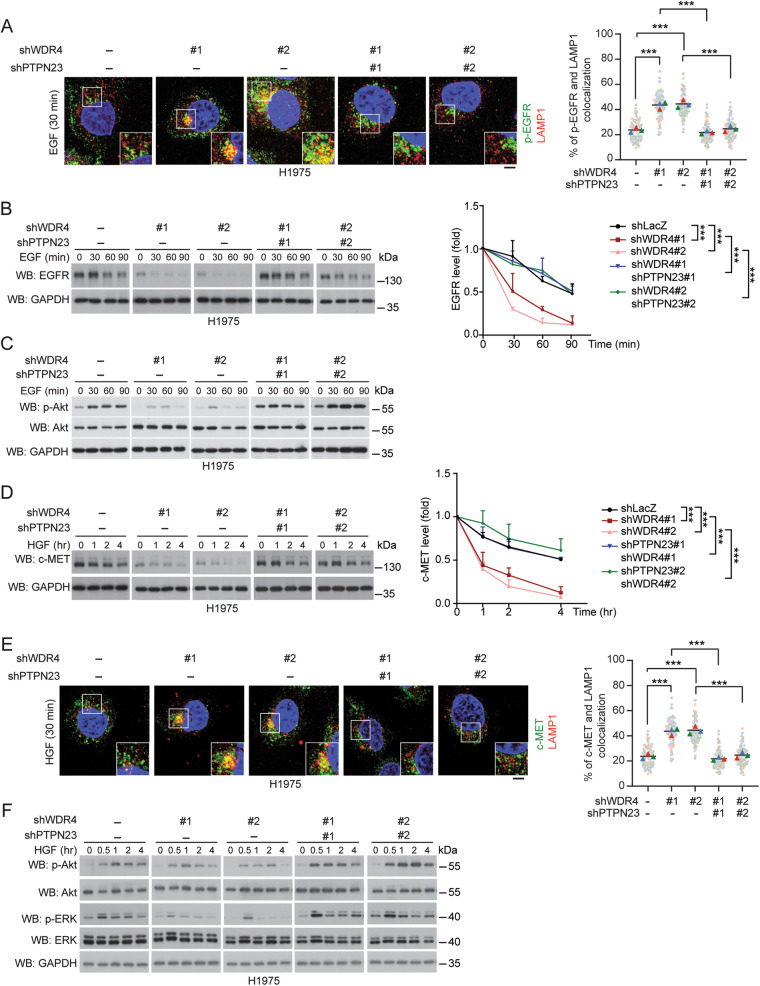
WDR4/PTPN23 axis inhibits EGFR mutant and c-MET lysosomal degradation to sustain their signaling. **A, E** Immunofluorescence staining for the colocalization of p-EGFR (**A**) or c-MET (**E**) with LAMP1 in H1975 cells stably expressing various shRNAs and treated with 100 ng/ml EGF or HGF for 30 min, respectively. The knockdown efficiencies of various shRNAs are shown in Supplementary Fig. S3A. Representative confocal images are shown on the left and quantitative data are on the right. Bar, 20 μm. Data are represented as mean, *n* = 3 (30 cells per group per experiment were counted.). *P*-values are determined by two-way ANOVA with Tukey’s post hoc test, ***P < 0.001. **B**, **D** Western blot analysis of EGFR or c-MET levels in cells as in (**A**) and treated with 100 ng/ml EGF or HGF for indicated time periods. The blots are representatives of three independent experiments and quantitative data are shown on the right. Data are mean ± SD, *n* = 3. *P*-values are determined by two-way ANOVA with Tukey’s post hoc test, ***P < 0.001. **C**, **F** Western blot analysis of indicated proteins in cells as in (**A**) and treated with 100 ng/ml EGF or HGF for indicated time periods.

Next, we extended our analysis to EGFR mutant. H1975 cells express an EGFR T790M/L858R mutant, which causes a resistance to 1st-generation EGFR TKI. We first established WDR4 knockdown and WDR4/PTPN23 double knockdown H1975 stable lines (Supplementary Fig. S3A). Importantly, WDR4 knockdown in H1975 cells enhanced p-EGFR trafficking to lysosomes in both EGFR-stimulated and unstimulated conditions (Fig. 4A and Supplementary Fig. S3B). This finding is consistent with an elevation of ligand-independent endocytosis observed from EGFR mutants, in comparison with wild type EGFR [24]. Furthermore, these effects of WDR4 knockdown on p-EGFR mutant trafficking were reverted by WDR4/PTPN23 double knockdown. Accordingly, WDR4 knockdown enhanced the turnover of EGFR mutant in EGF-treated cells, which was reversed by WDR4/PTPN23 double knockdown (Fig. 4B). The WDR4/PTPN23 axis also impeded the lysosomal degradation of EGFR mutant in EGF-untreated conditions (Supplementary Fig. S3C). Consistent with these findings, WDR4/PTPN23 axis promoted EGFR downstream signaling such as ERK and Akt phosphorylation in EGF-treated and untreated conditions in H1975 cells (Fig. 4C). Thus, our data indicate that WDR4/PTPN23 axis diminishes the lysosome trafficking and degradation of EGFR mutant in basal and ligand-stimulated conditions.

Besides EGFR mutation, c-MET amplification or mutation is an alternative mechanism to cause EGFR TKI resistance [13, 16]. Although c-MET undergoes ligand-induced endosome-lysosome trafficking similar to EGFR [43], the impact of PTPN23 on c-MET endocytic trafficking route has not been investigated. We showed that WDR4 knockdown in H1975 cells enhanced HGF-induced c-MET turnover and its colocalization with lysosome marker LAMP1, which were all reversed by WDR4/PTPN23 double knockdown (Fig. 4D, E). Consequently, c-MET downstream signaling such as ERK and Akt phosphorylation was suppressed by WDR4 knockdown and rescued by WDR4/PTPN23 double knockdown (Fig. 4F). The WDR4/PTPN23 axis also inhibited HGF-induced turnover of c-MET and sustained c-MET downstream signaling in H1299 cells (Supplementary Fig. S3D, E) and bafilomycin A1 treatment blocked the effect of WDR4/PTPN23 axis on HGF-induced c-MET turnover (Supplementary Fig S3F). In contrast, WDR4/PTPN23 axis did not affect *c-MET* mRNA and protein abundance in H1975 cells without HGF treatment (Supplementary Fig. S3G, H). Together, these findings uncover a role of WDR4/PTPN23 axis in suppressing lysosome trafficking and degradation of c-MET.

### WDR4/PTPN23 axis elicits multiple pro-tumor functions in NSCLC

**Fig. 5:**
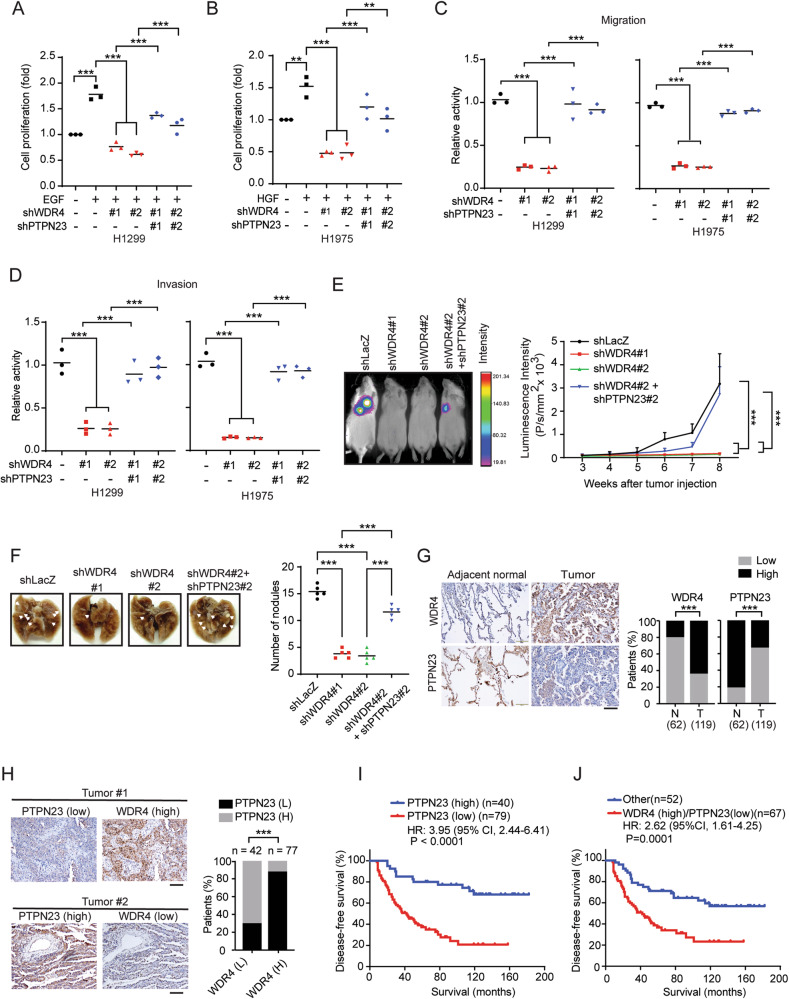
WDR4/PTPN23 axis promotes multiple cancer hallmarks and correlates with adverse prognosis in human lung cancer. **A**, **B** Cell proliferation assay of H1299 or H1975 cells stably expressing indicated shRNAs and stimulated with or without 100 ng/ml EGF (**A**) or HGF (**B**) for 24 hr, respectively. Data are represented as individual points and mean, *n* = 3. *P*-values are determined by one-way ANOVA with Sidak’s multi-comparison test, **P < 0.01, ***P < 0.001. **C**, **D** Migration and invasion assays of H1299 or H1975 cells stably expressing indicated shRNAs. Data are represented as individual points and mean, *n* = 3. *P*-values are determined by one-way ANOVA with Sidak’s multi-comparison test, ***P < 0.001. **E** Experimental metastasis assay for H1299 cells stably expressing indicated shRNAs. Representative bioluminescence images at week 8 and the kinetics of metastasis at indicated time points are shown on the left and right, respectively. Data are mean ± SD, *n* = 5. *P*-values are determined by two-way ANOVA with Tukey’s post-hoc test, ***P < 0.001. **F** Representative lung images at week 8 for the experiment shown in (**E**). Nodules are indicated by arrows and the number of metastatic nodules at the surface of lungs are shown on the right. Data are represented as individual points and mean, *n* = 5. *P*-values are determined by one-way ANOVA with Sidak’s multi-comparison test, ***P < 0.001. **G** Representative IHC images for WDR4 and PTPN23 expression in lung tumor tissues and their adjacent normal tissues (left). Bar, 20 µm. The summary of the WDR4 and PTPN23 expression profiles for 119 lung tumor tissues (**T**) and 62 adjacent normal tissues (**N**) are shown on the right. *P*-values are determined by Fisher’s exact test, ***P < 0.001. **H** Representative IHC images for 2 lung tumor tissues showing an inverse correlation for WDR4 and PTPN23 expression (left). Bar, 20 µm. The correlation of WDR4 expression with PTPN23 expression in 119 lung tumor specimens is shown on the right. *P*-values are determined by Fisher’s exact test, ***P < 0.001. **I**, **J** Kaplan-Meier analysis of lung cancer patients’ survival with the corresponding expression profiles. *P*-values are determined by log-rank test.

EGFR and c-MET pathways are critical for driving NSCLC malignancies. We thus evaluated the influence of WDR4/PTPN23 axis on various cancer hallmarks. First, WDR4 knockdown in H1299 and H1975 cells attenuated proliferation in EGF- and HGF-treated conditions, respectively, and these effects were reverted by WDR4/PTPN23 double knockdown (Fig. 5A, B). WDR4 knockdown also suppressed the ability of H1299 cells to form soft-agar colonies and tumor spheres (Supplementary Fig. S4A, B), the well-known assays for monitoring transformation ability and stemness property of cancer cells, respectively. These effects of WDR4 knockdown were abrogated by further knocking down PTPN23. The WDR4/PTPN23 axis also promoted migration and invasion of H1299 and H1975 cells (Fig. 5C, D). In addition, using an experimental metastasis model, we showed that WDR4-depleted H1299 cells exhibited a markedly decreased ability to form lung metastasis, in comparison with control cells and WDR4/PTPN23 double knockdown cells (Fig. 5E, F). Our study identifies a key role of WDR4/PTPN23 axis in promoting multiple malignant features of NSCLC.

### WDR4/PTPN23 axis is hyperactivated in human lung cancer and associated with poor prognosis

Next, we investigated the clinical relevance of WDR4/PTPN23 axis in lung cancer. Immunohistochemistry (IHC) analysis of 119 human lung cancer tissues and 62 adjacent non-tumor lung tissues revealed that WDR4 expression was elevated in tumor tissues compared with non-tumor tissues (Fig. 5G), consistent with our previous finding [38]. In contrast, PTPN23 expression was lower in tumors than in adjacent non-tumor tissues. We further observed a negative correlation between WDR4 expression and PTPN23 expression in tumor tissues (Fig. 5H). Furthermore, PTPN23 low expression and the combination of WDR4 high and PTPN23 low expression correlated with poor patient prognosis (Fig. 5I, J). Multivariate Cox regression analysis revealed that higher WDR4 expression and lower PTPN23 expression were independent poor prognostic factors in lung cancer, irrespective of other poor prognostic factor, such as the N stage (Supplementary Table S2). Thus, our data indicate the existence and hyperactivation of WDR4/PTPN23 axis in human lung cancer and the association of this axis with adverse prognosis.

### PTPN23 Bro40 peptide disrupts WDR4/PTPN23 interaction to promote EGFR and c-MET degradation

**Fig. 6:**
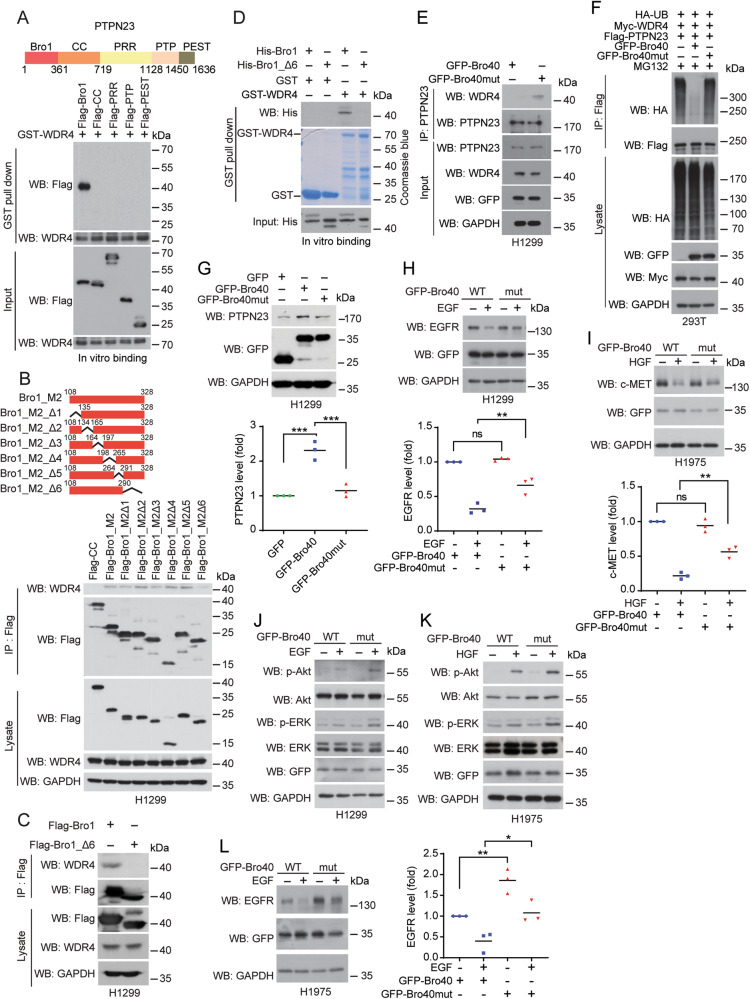
PTPN23 Bro40 disrupts WDR4/PTPN23 axis to downregulate EGFR and c-MET. **A** GST pull down analysis for mapping the PTPN23 domain involved in WDR4 interaction. Top: PTPN23 domain architecture. Bottom: The various Flag-PTPN23 segments purified from baculovirus were incubated with GST-WDR4 purified from bacteria. **B**, **C** Immunoprecipitation analysis of WDR4 interaction with PTPN23 Bro1 or its truncated mutants expressed in H1299 cells. **D** GST pull down analysis for the interaction between GST-WDR4 and Bro1 or Bro_Δ6 purified from bacteria. **E** Immunoprecipitation analysis for the interaction between endogenous PTPN23 and endogenous WDR4 in H1299 cells transiently transfected with GFP-Bro40 or GFP-Bro40mut and treated with 5 μM MG132 for 16 h. **F** Immunoprecipitation analysis of PTPN23 ubiquitination in 293T cells transiently transfected with indicated constructs. **G** Western blot analysis of PTPN23 levels in H1299 cells transiently transfected with the indicated constructs. Data are represented as individual points and mean, *n* = 3. *P*-values are determined by one-way ANOVA with Sidak’s multiple comparison test, ***P < 0.001. **H**, **I**–**L** Western blot analysis of EGFR or c-MET levels in H1299 or H1975 cells transiently transfected with GFP-Bro40 or GFP-Bro40mut and stimulated with 100 ng/ml EGF for 2 h or 100 ng/ml HGF for 4 h. The blots are representatives of three independent experiments and quantitative data are shown. Data are represented as individual points and mean, *n* = 3. *P*-values are determined by one-way ANOVA with Sidak’s multiple comparison test, *P < 0.05, **P < 0.01. ns: not significant. **J**, **K** Western blot analysis of indicated proteins in H1299 or H1975 cells transiently transfected with GFP-Bro40 or GFP-Bro40mut and stimulated with 100 ng/ml EGF or HGF for 4 h.

Given the high WDR4 expression and the low PTPN23 expression in lung cancers as well as the suppressive effect of WDR4/PTPN23 axis on EGFR and c-MET lysosomal degradation, we reasoned that disruption of the interaction between WDR4 and PTPN23 would stabilize PTPN23 to promote EGFR and c-MET trafficking to lysosome for degradation, thereby suppressing NSCLC malignancies and EGFR TKI resistance. To test this hypothesis, we first mapped the minimal region in PTPN23 responsible for binding WDR4. When each of the PTPN23 domains was expressed as a recombinant protein and purified from baculovirus, we found that only the Bro1 domain was pulled down by GST-WDR4 in vitro (Fig. 6A). Immunoprecipitation analysis further confirmed the interaction of PTPN23 Bro1 with WDR4 in vivo (Supplementary Fig. S5A). Within the Bro1 domain, a segment containing residues 108–328 (designated as M2) was capable of binding WDR4. Further mapping in this segment with various truncated mutants showed that deletion of the residues 289–328 (designated as Δ6) in the M2 segment or Bro1 domain abrogated their interactions with WDR4 in vivo (Fig. 6B, C) and in vitro (Fig. 6D). To further understand the binding mode between Bro1 domain and WDR4, a restraint-free docking was performed to generate the complex structure of WDR4-Bro1. Among the 10 best protein docking models obtained, 8 models showed that WDR4 bound to Bro1 at a region close to the M2–6 segment (Supplementary Fig. S5B), in agreement with our experimental data. Notably, the 40-residue M2–6 segment comprises an α-helix in which the 6 N-terminal residues (289–294) were predicted as a WDR4 contacting site (Supplementary Fig. S5C, top panel). Furthermore, the aspartic acid at residue 292 (D292) carries a negative charge while the surface of WDR4 counterpart is electrostatic positive, attracting each other to form a protein complex (Supplementary Fig. S5C, bottom panel). Finally, while WDR4 bound to the convex side of Bro1, all other known Bro1-binding partners, which govern various endocytic functions of PTPN23, occupied the concave side (Supplementary Fig. S5C, top panel), suggesting that disruption of the binding of WDR4 with PTPN23 would not affect the endocytic functions of PTPN23. We thus fused this 40-residue segment with GFP (termed GFP-Bro40) and also replaced the 289–294 residues with six alanine residues (termed GFP-Bro40mut) to test their influences on the interaction of WDR4 with PTPN23.

In line with the results of structural modeling analyses, expression of GFP-Bro40, but not GFP-Bro40mut, abrogated the endogenous interaction between WDR4 and PTPN23 (Fig. 6E). GFP-Bro40, however, did not affect PTPN23 binding to the ESCRT-0 subunit STAM2 (Supplementary Fig. S5D). Accordingly, GFP-Bro40, but not GFP-Bro40mut, greatly reduced WDR4-induced PTPN23 ubiquitination (Fig. 6F). GFP-Bro40 also elevated PTPN23 steady state level and prevented PTPN23 from degradation by proteasome (Fig. 6G and Supplementary Fig. S5E). Next, we tested the effects of GFP-Bro40 on EGFR and c-MET degradation and signaling. Compared to GFP-Bro40mut, GFP-Bro40 enhanced EGF-induced downregulation of EGFR in H1299 cells and HGF-induced downregulation of c-MET in H1975 cells (Fig. 6H, I). Consequently, ligand-induced activation of EGFR and c-MET downstream signaling was diminished in cells expressing GFP-Bro40, compared with those expressing GFP-Bro40mut (Fig. 6J, K). Furthermore, GFP-Bro40 expression in H1975 cells downregulated EGFR mutant in both EGF-stimulated and unstimulated conditions, compared with cells carrying GFP-Bro40mut (Fig. 6L). Thus, our study identifies the Bro40 peptide as a valuable agent to target the WDR4/PTPN23 axis for enhancing the degradation of EGFR, EGFR mutant, and c-MET, thereby desensitizing their downstream signaling.

### PTPN23 Bro40 peptide suppresses lung cancer malignancies and overcomes EGFR TKI resistance

**Fig. 7:**
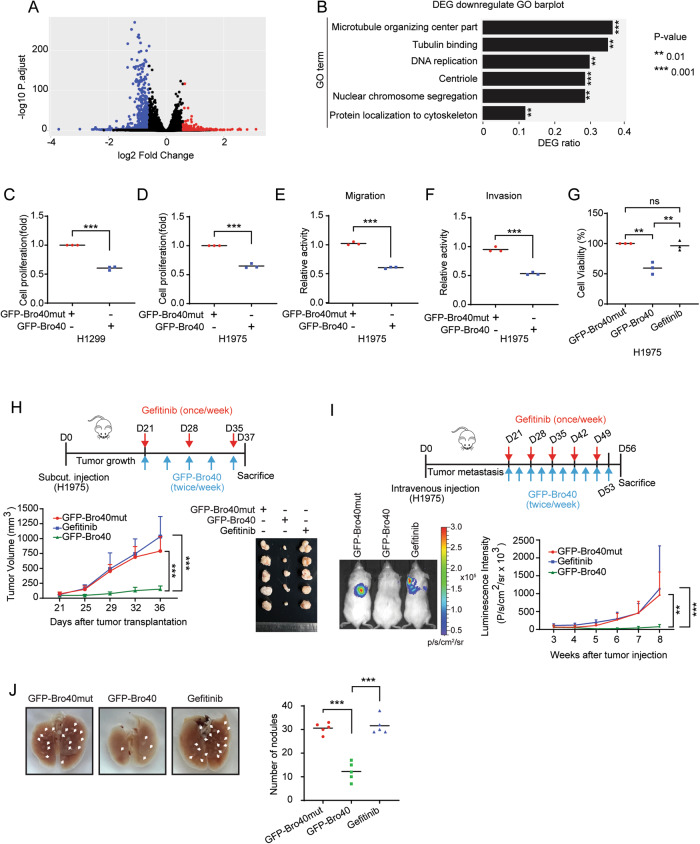
PTPN23 Bro40 suppresses lung cancer progression and overcomes EGFR TKI resistance. **A** Volcano plot for the comparison of RNA-seq data derived from H1299 cells expressing GFP-Bro40 and GFP-Bro40mut. DEGs are marked by blue and red dots. **B** GO analysis of genes that are downregulated by GFP-Bro40 in comparison with GFP-Bro40mut. **C**–**F** Cell proliferation, migration, and invasion analyses of H1299 or H1975 cells transiently transfected with GFP-Bro40 or GFP-Bro40mut. Data are represented as individual points and mean, *n* = 3. *P*-values are determined by two-sided Student’s *t*-test, ***P < 0.001. **G** MTT assay for H1975 cells transiently transfected with GFP-Bro40 or GFP-Bro40mut or treated with 1 µM gefitinib for 48 h. Data are represented as individual points and mean, *n* = 3. *P*-values are determined by one-way ANOVA with Tukey’s post hoc test, **P < 0.01. ns: not significant. **H** Mice that are subcutaneously transplanted with H1975 cells were treated with GFP-Bro40 or GFP-Bro40mut liposome nanoparticle or with gefitinib starting at day 21 after tumor cell implantation (Top panel). Tumor volumes were measured on the indicated days and plotted (bottom left panel). Data are mean ± SD, *n* = 5. *P*-values are determined by two-way ANOVA with Tukey’s post-hoc test, ***P < 0.001. Tumors were surgically removed on day 37 and their sizes are shown on the right. **I** Mice that were intravenously injected with H1975 cells were treated with GFP-Bro40 or GFP-Bro40mut liposome nanoparticle or with gefitinib as illustrated on the top panel. Representative images of bioluminescence analysis at day 56 after tumor cell injection are shown on the bottom left panel and the kinetics of metastasis are shown on the bottom right panel. Data are mean ± SD, *n* = 5. *P*-values are determined by two-way ANOVA with Tukey’s post-hoc test, **P < 0.01, ***P < 0.001. **J** Representative lung images on day 56 of the experiment shown in **I**. Nodules are indicated by arrows and the number of metastatic nodules at the surface of lungs are shown on the right. Data are represented as individual points and mean, *n* = 5. *P*-values are determined by one-way ANOVA with Sidak’s multi-comparison test, ***P < 0.001.

Next, we determined the impact of Bro40 peptide on the gene expression landscape by performing RNA-seq analysis on H1299 cells expressing GFP-Bro40 and GFP-Bro40mut. Using the criteria of fold change [GFP-Bro40/GFP-Bro40mut] > 1.5 or <0.66 and *P*adjust < 0.05 to define the differentially expressed genes (DEGs), we uncovered 225 upregulated and 779 downregulated genes from three biological repeats (Fig. 7A). Since downregulated genes are more numerous, we focused on this population. Gene Ontology (GO) analysis of this population revealed that microtubule organizing center, tubulin binding, DNA replication, centriole, nuclear chromosome segregation, and protein localization to cytoskeleton are among the enriched terms (Fig. 7B). This finding implies a suppressive role of Bro40 peptide in cell proliferation by impeding DNA replication and mitosis and in cell migration by compromising microtubule organizing function of centrosome and microtubule dynamics. In line with this idea, H1299 and H1975 cells expressing GFP-Bro40 exhibited a diminished cell proliferation, compared with cells carrying GFP-Bro40mut (Fig. 7C, D). GFP-Bro40 also decreased the proliferation of H1299 and H1975 cells in EGF- and HGF-treated conditions, respectively (Supplementary Fig. S6A, B). Furthermore, consistent with the downregulation of centrosomal proteins and tubulin binding proteins, H1299 and H1975 cells expressing GFP-Bro40 displayed a reduced microtubule polymerization ability, compared with cells expressing GFP-Bro40mut (Supplementary Fig. S6C, D). Accordingly, H1975 cells carrying GFP-Bro40 exhibited decreased migration and invasion abilities, compared with GFP-Bro40mut expressing cells (Fig. 7E, F). These data demonstrate the power of Bro40 peptide to induce multiple tumor suppressive effects in NSCLC.

Next, we focused on the anti-tumor effects of GFP-Bro40 on EGFR TKI-resistant H1975 cells and used gefitinib as a negative control. We found that GFP-Bro40 decreased the viability of H1975 cells, in comparison with GFP-Bro40mut and gefitinib (Fig. 7G). To determine the anti-tumor effect of GFP-Bro40 in vivo, we utilized the strategy of liposome-assisted delivery of GFP-Bro40 or GFP-Bro40mut expression construct into mice bearing H1975-derived tumors. We observed a marked suppression of tumor growth by GFP-Bro40 administration, compared with GFP-Bro40mut and gefitinib administration (Fig. 7H). Furthermore, IHC analysis of tumors derived from these mice revealed that GFP-Bro40 treatment downregulated EGFR, p-EGFR, c-MET and their downstream effectors p-Akt and p-ERK in tumor tissues, which were accompanied by a decreased tumor proliferation rate (determined by Ki67 staining) and an increased apoptosis (Supplementary Fig. S6E). Next, we tested the effect of GFP-Bro40 administration on the metastasis of H1975 cells using the experimental metastasis model. Compared with GFP-Bro40mut and gefitinib, GFP-Bro40 injection greatly suppressed metastasis, as revealed by bioluminescence analysis and visualization of lung nodules (Fig. 7I). Thus, GFP-Bro40 potentiates the degradation of EGFR mutant to block its downstream signaling in EGFR TKI-resistant NSCLC, thereby achieving potent anti-tumor and anti-metastasis effects.

**Fig. 8:**
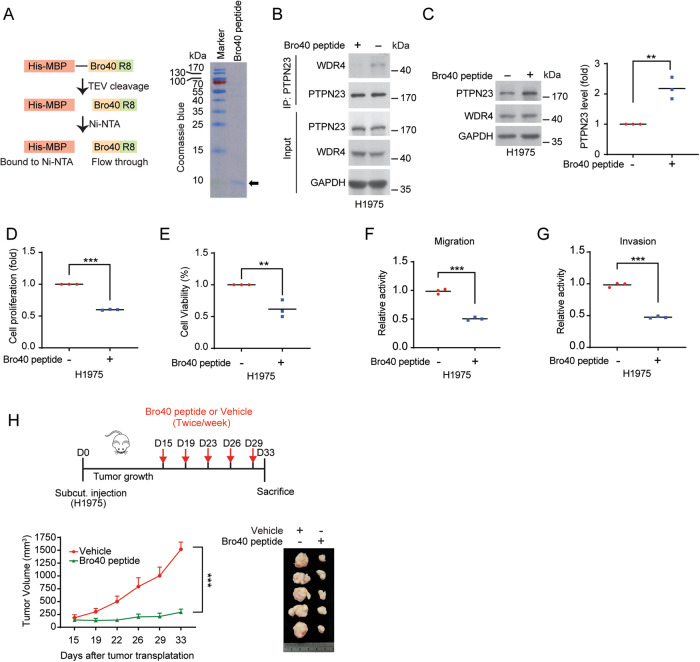
Cell penetrating Bro40 peptide blocks WDR4/PTPN23 interaction and overcomes EGFR TKI resistance. **A** Schematic representation of the generation of cell penetrating Bro40 peptide (left) and Coomassie blue staining of the Bro40 peptide (right). The position of Bro40 peptide is marked by an arrow. **B** Immunoprecipitation analysis for the interaction between endogenous PTPN23 and endogenous WDR4 in H1975 cells incubated with Bro40 peptide or vehicle and treated with 5 μM MG132 for 16 h. **C** Western blot analysis of PTPN23 levels in H1975 cells incubated with the Bro40 peptide. Data are represented as individual points and mean, *n* = 3. *P*-values are determined by two-sided Student’s *t*-test, **P < 0.01. **D**–**G** Cell proliferation, viability, migration, and invasion analyses of H1975 cells incubated with Bro40 peptide. Data are represented as individual points and mean, *n* = 3. *P*-values are determined by two-sided Student’s *t*-test, **P < 0.01, ***P < 0.001. **H** Mice that are subcutaneously transplanted with H1975 cells were treated with Bro40 peptide or vehicle at day 15 after tumor cell implantation (top). Tumor volumes were measured on the indicated days and plotted (bottom left). Data are mean ± SD, *n* = 5. *P*-values are determined by two-way ANOVA with Tukey’s post-hoc test, ***P < 0.001. Tumors were surgically removed on day 33 and their sizes are shown on the bottom right.

To directly assess the effect of Bro40 peptide on treating EGFR TKI-resistant NSCLC, we generated cell penetrating Bro40 peptide by fusing Bro40, along with 8 Arginine residues (R8), to the C-terminus of His-MBP. After the purification of this fusion protein, the His-MBP moiety was cleaved and the Bro40 peptide was purified (Fig. 8A). We found that administration of H1975 cells with cell penetrating Bro40 peptide disrupted the interaction between endogenous WDR4 and PTPN23 in H1975 cells and increased PTPN23 abundance (Fig. 8B, C). This Bro40 peptide also suppressed the proliferation, survival, migration, and invasion of H1975 cells (Fig. 8D–G). Furthermore, intratumoral injection of the Bro40 peptide greatly suppressed the growth of H1975-derived tumors in vivo (Fig. 8H). These data support the role of Bro40 peptide as a promising agent in treating EGFR TKI resistant NSCLC.

## Discussion

Although EGFR TKIs have been the standard treatment for NSCLC patients carrying EGFR mutations, development of acquired resistance is almost inevitable and becomes a major hurdle for disease management. Thus, there is an unmet need for developing new therapeutic strategies to overcome EGFR TKI resistance in NSCLC. In this study, we identify the ESCRT component PTPN23 as a substrate of WDR4-based Cul4 ubiquitin ligase. WDR4-mediated PTPN23 proteasomal degradation enhances the downstream signaling of wild type and mutant EGFR and c-MET by preventing these RTKs from lysosome trafficking and degradation. In keeping with these effects, WDR4/PTPN23 axis potentiates multiple malignant features of NSCLC. Clinically, WDR4/PTPN23 axis is hyperactivated in lung cancer and associated with poor prognosis. These findings point to a promise for targeting WDR4/PTPN23 axis to overcome EGFR TKI resistance. To prove this hypothesis, a PTPN23 peptide (Bro40) is designed to compete with endogenous PTPN23 for binding WDR4. We show that administration of this peptide to NSCLC not only stabilizes PTPN23 to reactivate the lysosomal degradation of wild type and mutant EGFR and c-MET, but also induces potent anti-tumor effects on EGFR TKI-resistant NSCLC cells to suppress their growth in vitro and in vivo, survival, migration, invasion, and metastasis. Thus, our study proposes a novel strategy for treating EGFR TKI-resistant NSCLC.

It has been evident that the gatekeeper EGFR mutants undergo altered endocytic pathways by enhancing the recycling and decreasing the degradation routes [24]. This provides an opportunity for redirecting EGFR mutants to the lysosomal degradation route as an anti-cancer strategy. Targeted protein degradation has emerged as an innovative concept for drug development. This concept utilizes an event-driven mode of action, which offers several advantages over conventional occupancy-driven pharmacology [18]. For instance, removal of the disease-causing proteins from cells could overcome drug resistance caused by mutations and prevent the accumulation of target proteins in cells from causing incomplete inhibition. In the case of EGFR, the former is a prevalent problem, while the latter is also a concern due to the existence of kinase-independent pro-tumor functions of EGFR [44–46]. Thus, elimination of EGFR protein is likely a more promising approach for achieving a durable and complete inhibition. The Bro40 peptide identified in this study potentiates the degradation of not only EGFR mutant but also c-MET, further expanding the potential utility of this peptide for combating EGFR TKI resistance. Notably, amplification or upregulation of several other RTKs, such as HER2, AXL, and IGF1R also contributes to the resistance to 1^st^- and 3^rd^-generation EGFR TKIs [17, 47]. Future studies will determine the influence of WDR4/PTPN23 axis on the lysosome trafficking and degradation of these RTKs.

PTPN23 is a large protein with multiple domains and binding partners [31]. Its Bro1 domain is responsible for binding ESCRT-0 subunit STAM2, ESCRT-III subunit CHMP4, and endosome proteins SARA and endofin. Notably, these proteins share a common binding pocket in the Bro1 domain and their competitive bindings are implicated in the sequential actions for endosome recruitment of PTPN23, ESCRT-0-dependent cargo sorting, and cargo transferring to ESCRT-III [28, 29, 48]. WDR4, however, binds to an opposite surface on the Bro1 domain in relation to these endocytic proteins. This would allow the utilization of WDR4-binding region, i.e., the Bro40 peptide, as an agent for disruption of the binding between endogenous WDR4 and endogenous PTPN23 without affecting PTPN23 endocytic functions. Accordingly, our study shows that Bro40 does not affect PTPN23 binding to STAM2. Despite the demonstration of this layer of specificity, we cannot rule out the possibility that Bro40 affects the cellular functions of other Bro1 domain-containing proteins. Further studies are required for fully characterizing the specificity and safety of this Bro40 peptide.

Besides promoting the lysosome trafficking of several oncogenic cell surface receptors, PTPN23 suppresses other oncogenic pathways such as Src and Yap [49]. Accordingly, the Bro40 peptide downregulates not only EGFR and c-MET protein abundance, but also the expression of a set of genes involved in DNA replication, mitosis, microtubule dynamics and centrosome function. Despite the mechanism for their reduced expression warrants further investigation, this effect is consistent with the inhibitory roles of Bro40 in cell proliferation and migration. Thus, gene expression alteration might also contribute to the anti-tumor effects of Bro40 peptide.

The endosome-lysosome pathway plays a key role in regulating the signaling output and duration of oncogenic cell surface receptors, such as RTKs and integrins, to influence on cancer malignancies. Reciprocally, cancer cells often display dysregulated endocytic trafficking to bias the recycling of RTKs and integrins [50] and several underlying mechanisms have been identified recently. First, dynamin 1, a neuron-enriched dynamin isoform, is activated in NSCLC through the Akt/GSK3β signaling pathway and this activation alters clathrin-mediated endocytosis by increasing the initiation of clathrin-coated pits (CCP) assembly but impairing CCP maturation, resulting in an increased cargo recycling [51]. In addition, the clathrin light chain isoform b (CLCb) is upregulated in NSCLC to increase EGFR endocytosis and recycling through a dynamin 1-dependent mechanism [52]. The ERK substrate FCHSD2 also impacts on RTKs endocytic trafficking by enhancing their recycling and decreasing their lysosomal degradation [53, 54]. In this study, we identify a novel mechanism by which NSCLC cells skew endocytic trafficking of RTKs, such as EGFR and c-MET. That is, the upregulation of WDR4-based ubiquitin ligase in NSCLC enhances the degradation of ESCRT protein PTPN23, thereby impairing the late endosome/lysosome trafficking of RTKs to result in a sustained ERK and Akt signaling. Given that ERK and Akt signaling could induce FCHSD2 and CLCb/dynamin 1 to further distort the endocytic pathway, WDR4/PTPN23 axis might participate in a feedforward mechanism to coordinate with these endocytic regulators for a robust redirection of oncogenic cell surface receptors towards a recycling fate. In this way, disruption of the WDR4/PTPN23 axis would play a pivot role in promoting the lysosomal degradation of these receptors.

In sum, this study identifies an ubiquitin/proteasome-dependent pathway that impedes lysosome trafficking and degradation of RTKs, such as EGFR and c-MET. Targeting this pathway holds a promise to overcome EGFR TKI resistance in NSCLC.

## Materials and methods

### Cell culture and transfection

HEK293T, 293FT, H1299, and A549 cells were obtained from ATCC and cultured in Dulbecco’s Modified Eagle’s medium (DMEM) supplemented with 10% fetal bovine serum (FBS) and 1 % penicillin/streptomycin (P/S) (Gibco). H1975 cells were kindly provided by Ching-Chow Chen (National Taiwan University, Taiwan) and cultured in RPMI-1640 medium supplemented with 10% FBS and 1% P/S. Lipofectamine 2000 (Invitrogen), TransIT-X2 (Mirus Bio), and calcium phosphate method were used for transfection.

### Plasmids

Plasmids for pRK5-based WDR4, WDR4 R219A, Cul4A, Cul4B, ROC1, DDB1, and HA-ubiquitin were described previously [38]. WDR4 cDNA was amplified by PCR and cloned to pGEX-4T-1. Constructs for GFP-Rab11 and PTPN23 were gifts from Guang-Chao Chen (Academia Sinica, Taiwan). PTPN23 cDNA was amplified and subcloned to pRK5 or pVL1392 with a Flag-tag or His-tag. The various PTPN23 mutants were generated by PCR or site-directed mutagenesis and cloned to pRK5, pVL1392, and/or pEGFP-N3.

### Antibodies and reagents

All antibodies used in this study are described in Supplementary Table S3. Cycloheximide, bafilomycin A1, and MG132 were from Sigma-Aldrich, whereas gefitinib was purchased from MedChem Express. EGF and HGF were from Sino Biological.

### SILAC-labeling, cell lysis, and protease digestion

Cells were cultured for more than 5 doublings in DMEM deficient in L-Arginine and L-Lysine (Thermo Fisher Scientific) supplemented with 10% dialyzed FBS (Biological Industries), 1% P/S, and L-Arginine-HCl [^13^C_6_, ^15^N_4_] (Arg-10)/L-Lysine-HCl [^13^C_6_] (Lys-6), or L-Arginine (Arg-0)/L-Lysine (Lys-0) (Thermo Fisher Scientific). Cells were treated with 5 μM MG132 for 2 h before harvest. The cell pellets were lysed at room temperature in urea lysis buffer containing 8 M urea, 50 mM Tris-HCl (pH 8.0), 150 mM NaCl, 1 mM EDTA, and protease inhibitor (Roche). The lysates were cleared by centrifugation at 14,800 rpm for 10 min and lysates containing 15 mg proteins per sample were mixed. Proteins were reduced with 5 mM DTT at 37°C for 45 min and subsequently alkylated with 10 mM iodoacetamide at room temperature for 30 min in the dark. Lysates were diluted to 4 M urea with 50 mM Tris-HCl (pH 8.0), and proteins were digested with Lys-C (Wako) at 37°C for 4 h. The peptide mixtures were further diluted to 1 M urea and digested with Trypsin (Promega) at 37°C for overnight. The reaction was quenched with formic acid, desalted by Sep-Pak PlusC18 cartridge (Waters), and lyophilized.

### Fractionation for proteome analysis

Dried peptides were reconstituted in 0.1% trifluoroacetic acid (TFA) and then loaded onto an equilibrated, high-pH, reversed-phase fractionation spin column. Peptides were bound to the hydrophobic resin under aqueous conditions and desalted by washing the column with water. Bound peptides were eluted into eight fractions with a step gradient of increased acetonitrile concentration.

### Enrichment of K-ε-GG peptides for ubiquitylome analysis

K-ε-GG peptides were enriched by PTMScan Ubiquitin Remnant Motif Kit (Cell Signaling Technology) according to manufacturer’s instructions. Briefly, peptides were reconstituted in immunoaffinity purification (IAP) buffer and incubated with PTMScan Motif Antibody conjugated to protein A agarose beads at 4°C for overnight. Beads were washed twice with 1.5 ml ice-cold IAP buffer followed by three washes with ice-cold 2dH_2_O. K-ε-GG peptides were eluted with 0.15% TFA twice and desalted by C18 Stage Tip (Millipore).

### LC-MS/MS

NanoLC−nanoESi-MS/MS analysis was performed on a Thermo UltiMate 3000 RSLCnano system connected to an Thermo Orbitrap Fusion mass spectrometer (Thermo Fisher Scientific, Bremen, Germany) equipped with a nanospray interface (New Objective, Woburn, MA) and followed procedures as previously described [55]. Briefly, peptide mixtures were loaded onto a 75 μm ID, 25 cm length PepMap C18 column (Thermo Fisher Scientific) packed with 2 μm particles with a pore width of 100 Å and were separated using a segmented gradient in 120 min from 5 to 35% solvent B (0.1% formic acid in acetonitrile) at a flow rate of 300 nl/min. Solvent A was 0.1% formic acid in water. The mass spectrometer was operated in the data-dependent mode. Briefly, survey scans of peptide precursors from 350 to 1600 *m*/*z* were performed at 120 K resolution with a 2 × 10^5^ ion count target. Tandem MS was performed by isolation window at 2 Da with the quadrupole, CID fragmentation with normalized collision energy of 30, and rapid scan MS analysis in the ion trap. The MS^2^ ion count target was set to 10^4^ and the max injection time was 50 ms. Only those precursors with charge state 2–6 were sampled for MS^2^. The instrument was run in top speed mode with 3 s cycles; the dynamic exclusion duration was set to 60 s with a 10 ppm tolerance around the selected precursor and its isotopes. Monoisotopic precursor selection was turned on. For K-ε-GG proteome analysis, the survey scans of peptide precursors from 350 to 1600 *m/z* were performed at 60 K resolution with a 2 × 10^5^ ion count target. Precursor were fragmented by high energy collision-induced dissociation (HCD) at normalized collision energy of 30, and analyzed using the Orbitrap. The MS^2^ ion count target was set to 5 × 10^4^ and the max injection time was 100 ms.

The K-ε-GG peptides were identified with the SEQUEST database search algorithm implemented in the Proteome Discoverer (v2.2) (Thermo Scientific, San Jose, CA). The enzyme specificity was set to trypsin (full) allowing for two missed cleavages and precursor mass tolerances were 10 ppm for parent ions and 0.2 Da for fragment ions. Dynamic modifications were set for heavy lysine (^13^C_6_) (+ 6.02013), heavy arginine (^13^C_6_^15^N_4_) (+10.00827), methionine oxidation (+15.99492 Da), lysine ubiquitination (+114.04293 Da/+383.22810), asparagine and glutamine deamidation (+0.98402 Da) and protein N-terminal acetylation (+42.03670). A maximum of 3 dynamic modifications was allowed per peptide and a static modification of +57.02147 Da was set for carbamidomethyl cysteine. The Percolator node within Proteome Discoverer was used to filter the peptide spectral match (PSM) false discovery rate to 1% and peptide areas were calculated using the Precursor Ions Quantifier node. The unique and razor peptides were used for protein quantitation. Datasets were normalized on total peptide amount and precursor intensity abundances were subsequently used to compare the proteins and peptides across the replicates and datasets.

### RNA interference and generation of CRISPR KO cell line

Lentivirus-based shRNA constructs were obtained from National C6 RNAi Core Facility (Taipei, Taiwan). The target sequences of various shRNAs are listed in Supplementary Table S4. WDR4 KO cells were generated by National C6 RNAi Core Facility. Briefly, two sgRNAs were designed to target exon 1 and exon 7. The sgRNAs were cloned to pAll-Cas9.Ppuro (National C6 RNAi Core Facility). The targeting sequences for exon 1 are: 5'GTCACCGACCGGTGCGGAC & 5'GCCGCGTCTCTGCGCCTGGA and for exon 7 are: 5'TGTGGGGCTTCCGTCAGGT & 5'GGCAGTCGCCTGACTTGAG. A549 cells were transfected with the generated plasmids, selected with puromycin, and followed by single cell colony isolation. The devoid of WDR4 expression was confirmed by Western blot.

### Western blot and immunoprecipitation

Cells were lysed with RIPA buffer containing 50 mM Tris-HCl (pH 7.5), 150 mM NaCl, 1% NP-40, 1% sodium deoxycholate, 0.1% SDS, 1 mM PMSF, 1 µg/ml aprotinin, and 1 µg/ml leupeptin. Lysates containing equal amounts of proteins were subjected to immunoprecipitation and Western blot analyses as described previously [56].

### In vitro ubiquitination assay

WDR4-based Cul4 ligase complex was purified with anti-Myc beads (Sigma-Aldrich) from HEK293T cells cotransfected with Myc-Cul4A or Myc-Cul4B, together with other complex subunits. His-PTPN23 was purified from baculovirus by incubating lysates with Ni-Sepharose beads (Cytiva) and eluted with imidazole. The E3 ligase complex bound on Myc beads was incubated at 37 °C for 2 h in 20 μl reaction mixture containing 40 ng yeast E1, 500 ng E2 (UbcH5a), 300 ng His-PTPN23 and other components as described previously [57]. The E1, E2, and His-ubiquitin were purchased from R&D systems.

### In vitro binding assay

In vitro binding assay was performed as described previously [38]. Briefly, GST-WDR4 bound on the glutathione beads (GE Healthcare) was incubated with His-PTPN23 or Flag-PTPN23 purified from baculovirus in binding buffer containing 50 mM Tris-HCl (pH 7.5), 150 mM NaCl, and 1% NP-40. The beads were washed in the washing buffer containing 1 M Tris-HCl (pH 7.5), 0.5% Triton X-100, and 150 mM NaCl and the bound proteins were analyzed by Western blot.

### Immunofluorescence

Cells grown to 70% confluency were washed with PBS, fixed with 4% paraformaldehyde for 15 min at room temperature and permeabilized with 0.1% Triton X-100 in PBS for 10 min. Cells were blocked with 5% goat serum and 1% BSA in PBS for 1 h at room temperature and incubated with primary antibody in PBS (with 5% goat serum and 1% BSA) at 4 °C for overnight. Cells were then washed three times with 0.1% Triton X-100 in PBS at room temperature, followed by incubation with secondary antibody for 1 h. Nuclei were stained with UltraCruz (Santa Cruz). Images were acquired by using an Olympus FV3000 Confocal Microscope with a 60×/1.40 oil objective lens (UPlanSApo). To quantify the colocalization of puncta, green and red channels were stacked together by using Image J 1.53c. The yellow regions were considered as colocalization and the area was calculated by the Measure function of Image J 1.53c.

### EGF internalization assay

Cells grown in culture medium to 70% confluency were starved in serum-free medium for 16 h and then transferred to 4 °C prior to the addition of 50 ng/ml biotinylated EGF (Invitrogen). Cells were washed 3 times with ice-cold PBS to remove unbound biotinylated EGF and then incubated at 37 °C for various time periods to allow EGF internalization. Biotinylated EGF not yet internalized was blocked with free avidin (0.05 mg/ml, Invitrogen) on ice and washed 3 times with ice-cold PBS to remove the unbound avidin. The internalized EGF was analyzed by Human EGF ELISA Kit (Invitrogen) according to manufacturer’s instruction, followed by absorbance measurement at 450 nm with Infinite M1000 pro (Tecan).

### RT-qPCR

Cells were lysed with TRIzol™ Reagent (Thermo Fisher Scientific), followed by chloroform separation, isopropanol precipitation and ethanol dehydration. An equal amount of RNA was reverse transcribed by iSript cDNA Synthesis Kit (Bio-Rad). Real-time PCR was performed on an ABI 7500 Fast Real-Time PCR system with Power SYBR Green PCR Master Kit (Applied Biosystem). GAPDH was used as an internal control. The sequences of PCR primers are listed in Supplementary Table S5.

### RNA-seq

RNA quality control and sequencing were performed by BioTools, Taiwan. RNA quality was maintained at above 7.2 over 10 followed by a 20 million reads per sample. Sequencing libraries were generated using KAPA mRNA HyperPrep Kit (KAPA Biosystems, Roche, Basel, Switzerland) following manufacturer’s recommendations and index codes were added to attribute sequences to each sample. The original data obtained by high-throughput sequencing using Illumina NovaSeq 6000 platform were transformed into raw sequenced reads by CASAVA base calling and stored in FASTQ format. FastQC and MultiQC were used to check fastq files for quality. The obtained raw paired-end reads were filtered by Trimmomatic to discard low-quality reads, trim adaptor sequences, and eliminate poor-quality bases with the following parameters: LEADING:3 TRAILING:3 SLIDINGWINDOW:4:15 MINLEN:30. The obtained high-quality data (clean reads) were used for subsequent analysis. Read pairs from each sample were aligned to the reference genome (H. sapiens, GRCh38) by the HISAT2 software. FeatureCounts was used to count the reads numbers mapped to individual genes. For gene expression, the “Trimmed Mean of M-values” normalization (TMM) was performed using DEGseq without biological duplicate and the “Relative Log Expression” normalization (RLE) was performed using DESeq2 with biological duplicate. DEGs analysis of two conditions was performed in R using DEGseq (without biological replicate) and DESeq2 (with biological replicate), which based on negative binomial distribution and Poisson distribution model, respectively. The resulting p-values were adjusted using the Benjamini and Hochberg’s approach for controlling the FDR. Finally, bioinformatics analyses were performed by Biotools RNA-seq Platform.

### Migration and invasion assays

Migration and invasion assays were performed by Transwell polycarbonate membrane cell culture inserts (8-µm pore size, EMD Millipore). For migration assay, 3 ×10^4^ cells were resuspended in serum-free medium and plated onto the upper chamber, and medium containing 10% FBS was added in the lower chamber. For invasion assay, the upper side of the Transwell membrane was coated with Matrigel. 3 × 10^4^ cells in serum-free medium were plated onto the upper chamber. Cells were incubated at 37 °C for 10 h. Cells that migrated onto the lower membrane surface were fixed with 4% formaldehyde, stained with DAPI, and counted.

### Soft agar colony formation assay

5×10^3^ cells were mixed with 0.05% noble agar/DMEM medium and plated onto 6-well plates. The agar was covered by 1 ml culture medium. Colony formation was measured at day 21.

### PLA

PLA was performed with Duolink^TM^ In Situ Detection Reagents (Sigma-Aldrich) according to manufacturer’s protocol.

### Cell viability and proliferation assays

Cell viability was analyzed by MTT assay. Briefly, cells were seeded onto 96-well plates at a concentration of 5×10^3^ cells/well. The next day, cells were treated with 0.4 mg/ml MTT reagent (Methyl thiazolyl diphenyl tetrazolium bromide; MedChemExpress) for 4 h. After addition of DMSO to the cells, absorbance was measured at 590 nm.

Cell proliferation was assayed by BrdU Cell Proliferation Assay Kit (Merck- Millipore) according to manufacturer’s protocol. Briefly, cells were seeded onto 96-well plates and treated with various reagents. The next day, cells were treated with BrdU reagent for 4 h and fixed for determining BrdU incorporation.

### Tumor sphere formation

Tumor sphere formation assay was performed as described previously [58]. Briefly, cells were seeded onto 6 well ultra-low attachment plates at a density of 1×10^3^ cells/well. Images were taken after 4 days by a Nikon Eclipse Ti microscope.

### IHC

Primary lung cancer tissue specimens and microarrays were obtained from the Tissue Bank of National Cheng Kung University Hospital. A total of 119 tumor tissues and 62 adjacent non-tumor tissues were analyzed. Tumor sections derived from xenograft models were prepared by Pathology Core Facility, Institute of Biomedical Sciences (Academia Sinica, Taiwan). IHC staining was performed with Novolink Max Polymer kit (Leica Biosystems, Wetzlar, Germany). Slides were dewaxed with xylene/ethanol and antigen retrieval was performed with TRS buffer in a microwave oven. After blocking, the slides were incubated with primary antibodies. The peroxidase activity was visualized with diaminobenzidine tetrahydroxychloride (DAB) solution. The sections were counter stained with hematoxylin.

### Animal experiments

All mouse experiments were conducted according to the guidelines of animal ethical regulations and approved by the Experimental Animal Committee, Academia Sinica, Taiwan. In all experiments, animals were randomized and blindly allocated to experimental groups and no sample was excluded from the analyses. However, the investigators were not blinded to allocation during experiments. For experimental metastasis model, 1 × 10^6^ cells tagged with luciferase were resuspended in 100 µl PBS and injected intravenously into 8-week-old male NOD.Cg-Prkdc^scid^ /J mice (BioLASCO Co., Taiwan). In certain experiments, gefitinib (100 mg/kg) was injected intraperitoneally once a week, whereas plasmid encoding GFP-Bro40 or GFP-Bro40mut (0.75 mg/kg) incubated with the in vivo jetPEI delivery reagent (PEI; Polyplus Transfection) was intravenously injected twice a weak. Lung metastasis was monitored by bioluminescence imaging using a PerkinElmer in vivo imaging system (IVIS).

For assaying tumor growth, 8-week-old male NOD.Cg-Prkdc^scid^ /J mice were subcutaneously inoculated with 1 × 10^6^ cells. Gefitinib and plasmid encoding GFP-Bro40 or GFP-Bro40mut were injected as described above. Alternatively, Bro40 peptide (2 mg/kg) or buffer alone was injected intratumorally twice a week. Tumors volumes were measured every 3 or 4 days by the equation: ${{\rm{mm}}}^{3}={\rm{\pi }}/6\times [{\rm{length}}({\rm{mm}})]\times {[{\rm{width}}({\rm{mm}})]}^{2}$.

### In silico protein docking

The protein structures of WDR4 (ID: 2VDU), Bro1 (ID: 5MJZ), Bro1/CHMP4B (ID: 5MK2), Bro1/endofin (ID: 5MK0), Bro1/SARA (ID: 5MJY) and Bro1/STAM2 (ID: 5CRV) were obtained from Protein Data Bank (PDB) with denoted IDs for the structural analysis. The docking model of WDR4 and Bro1 was generated by the ClusPro 2.0 web server (https://cluspro.bu.edu/publications.php). The WDR4-Bro1 structure aligned with listed Bro1 domain structures was analyzed with educational PyMol 2.5.2 (Schrödinger, LLC).

### Microtubule regrowth assay

3 ×10^5^ cells were seeded on coverslip overnight. Cells were incubated at 4 °C for 1 h to depolymerize microtubules. To re-polymerize microtubules, cells were incubated in humidified atmosphere with 5% CO_2_ at 37 °C for 10 min. Cells were then washed with ice-cold PBS and fixed by 100% ice-cold methanol. Cells were blocked with 3% BSA in PBST (PBS with 0.1% Trion X-100) for 30 min and incubated with primary antibody at room temperature for 2 h. Next, cells were washed with PBST for 3 times followed by incubation with secondary antibody for 1 h. Nuclei were stained with UltraCruz and images were acquired by using Model Zeiss LSM700 Confocal Microscopy with a 100×/1.40 oil objective lens (UPlanSApo).

### Generation of cell-penetrating Bro40 peptide

Bro40 peptide sequences with 8 arginine residues were cloned to pKWu-135-pRK5FDuet1-His8-MBP plasmid. The plasmid was transformed into the *E. coli Rosetta2* and the bacteria were cultured in LB. After fusion protein induction by 0.6 mM IPTG, bacterial cells were lysed by lysis buffer (20 mM HEPES, 300 mM NaCl, 10 mM imidazole, 10 mM β-ME) supplemented with 200 mM PMSF. The supernatant was incubated with Ni-NTA Sepharose resin (Cytiva) for 1 h at 4 °C and then loaded onto a column. After washing the column sequentially with wash buffer (20 mM HEPES pH 7.5, 300 mM NaCl, β-ME) supplemented with 10 mM and 20 mM imidazole, His-tagged protein was eluted with elution buffer (20 mM HEPES pH 7.5, 300 mM NaCl, β-ME, 600 mM imidazole), and then dialyzed in dialysis buffer (20 mM HEPES pH 7.5, 300 mM NaCl, β-ME) overnight and treated with TEV protease (1 mg TEV protease/50 mg of protein) for overnight at 4 °C. The protein solution was incubated with Ni-NTA Sepharose beads for 1 h at 4 °C to remove His-MBP-TEV fragment and the flow through was recovered. The peptide was quantified by measuring the absorbance at A280.

### Statistical analyses

For each experiment, sample size was chosen based on similar experimental approaches reported in the literatures and no data were excluded from the analysis. Two-sided, unpaired Student’s *t*-test was used for the comparisons between 2 groups, and ANOVA was used for multigroup comparisons. Fisher’s exact test was applied for the analysis of clinicopathological data. Kaplan-Meier estimation and log-rank test were used to compare the patient survival curves. Cox regression analysis was used for univariate and multivariate analyses. All statistical analyses were performed using GraphPad Prism 8 software. P value less than 0.05 was considered as statistically significant.

### Supplementary information

Supplementary Figures and Tables

Checklist

original data file

## Data Availability

The original Mass Spectrometry data from ubiquitylome and proteome analysis are deposited to the ProteomeXchange Consortium via PRIDE partner repository with the project accession number PXD025361. The RNA-seq data are deposited to the GEO database with the accession number GSE212016.
