# Correction: PTPN23 ubiquitination by WDR4 suppresses EGFR and c-MET degradation to define a lung cancer therapeutic target

**DOI:** 10.1038/s41419-024-06747-x

**Published:** 2024-07-02

**Authors:** Shaifali Singh, Nai Yang Yeat, Ya-Ting Wang, Shu-Yu Lin, I-Ying Kuo, Kuen-Phon Wu, Won-Jing Wang, Wen-Ching Wang, Wu-Chou Su, Yi-Ching Wang, Ruey-Hwa Chen

**Affiliations:** 1 Institute of Biological Chemistry, Academia Sinica, Taipei, 115 Taiwan; 2 Chemical Biology and Molecular Biophysics Program, Taiwan International Graduate Program, Academia Sinica, Taipei, 115 Taiwan; 3 Institute of Molecular & Cellular Biology and Department of Life Science, National Tsing Hua University, Hsinchu, 300 Taiwan; 4 Department of Chemistry, National Tsing Hua University, Hsinchu, 300 Taiwan; 5 Department of Pharmacology, College of Medicine, National Cheng Kung University, Tainan, 701 Taiwan; 6 Institute of Basic Medical Sciences, College of Medicine, National Cheng Kung University, Tainan, 701 Taiwan; 7 Institute of Biochemistry and Molecular Biology, National Yang Ming Chiao Tung University, Taipei, 112 Taiwan; 8 Division of Oncology, Department of Internal Medicine, National Cheng Kung University Hospital, College of Medicine, National Cheng Kung University, Tainan, 701 Taiwan

**Keywords:** Cancer, Cancer therapy, Ubiquitylation

Correction to: *Cell Death and Disease* 10.1038/s41419-023-06201-4, published online 11 October 2023

In the ‘Data Availability’ section, the project accession number was corrected from PXD494674 to PXD025361. The original article has been corrected.

