# Supplementary material for: PTPN23 ubiquitination by WDR4 suppresses EGFR and c-MET degradation to define a lung cancer therapeutic target
Source: Cell Death Dis. 2023 Oct 11;14(10):671. doi: 10.1038/s41419-023-06201-4 (PMC10567730; doi:10.1038/s41419-023-06201-4)
Supplement: Supplementary file 1 — Supplementary Figures and Tables [file 41419_2023_6201_MOESM1_ESM.docx]

**Supplementary Materials**

**PTPN23 ubiquitination by WDR4 suppresses EGFR and c-MET degradation to define a lung cancer therapeutic target**

**Supplementary Figures S1-S6**

**Supplementary Tables S1-S5**

**
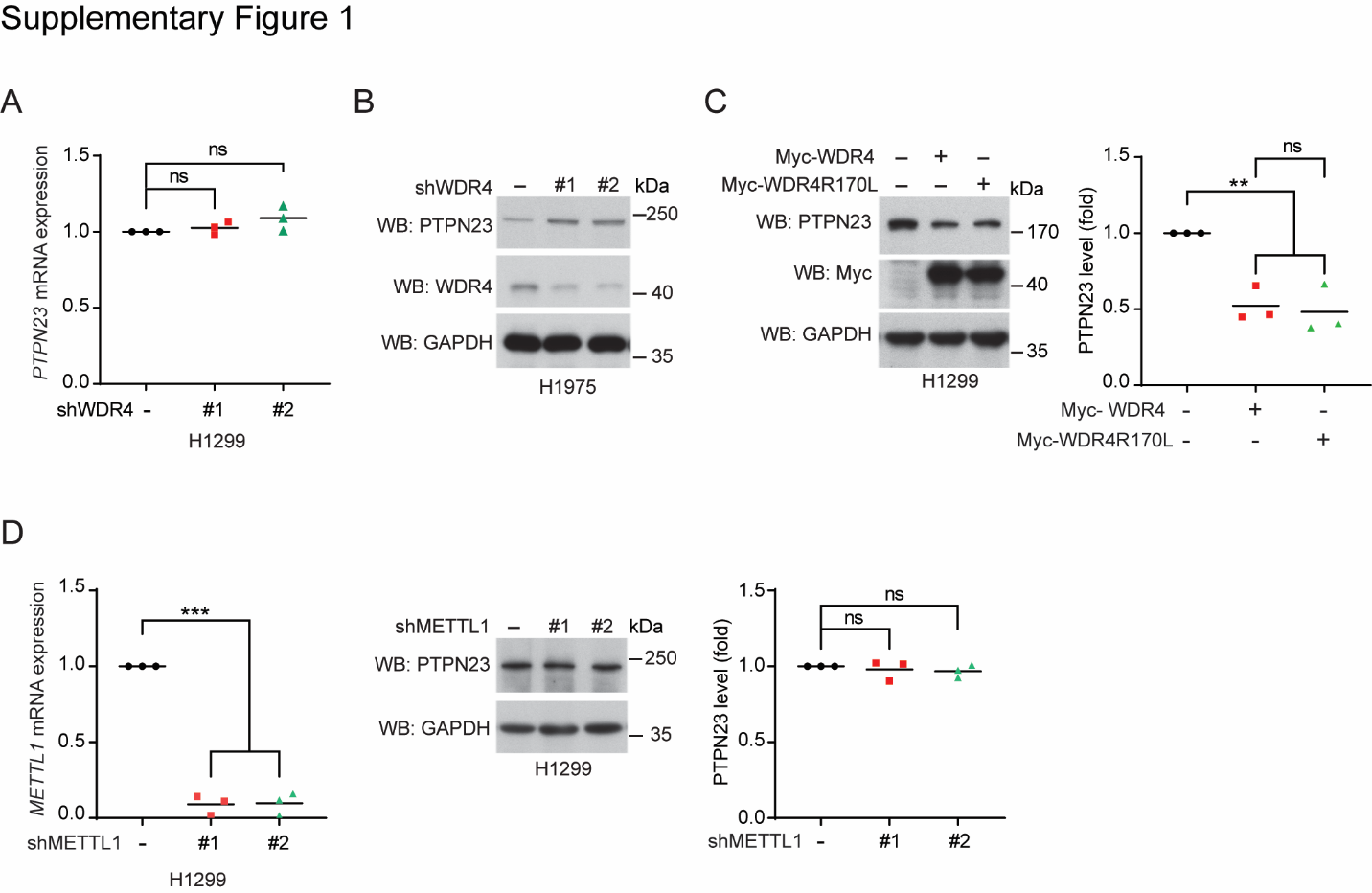
**

**Supplementary Figure S1. WDR4 depletion elevates PTPN23 protein, but not mRNA level.** (A) RT-qPCR analysis of *PTPN23* mRNA expression levels in H1299 cells stably expressing indicated shRNAs. Data are represented as individual points and mean, n=3. P values are determined by one-way ANOVA with Tukey’s post-hoc test. ns: not significant. (B) Western blot analysis of indicated proteins in H1975 cells stably expressing WDR4 shRNAs. (C) Western blot analysis of PTPN23 expression levels in H1299 cells transfected with indicated constructs. The blots are representatives of three independent experiments and quantitative data are shown on the right. (D) Western blot analysis of PTPN23 expression levels in H1299 cells stably expressing indicated shRNAs. The knockdown efficiency of METTL1 shRNAs are shown on the left. The blots are representatives of three independent experiments and quantitative data are shown on the right. In (C) and (D), data are represented as individual points and mean, n=3. P values are determined by one-way ANOVA with Tukey’s post-hoc test. **P<0.01, ***P<0.001, ns: not significant.

**
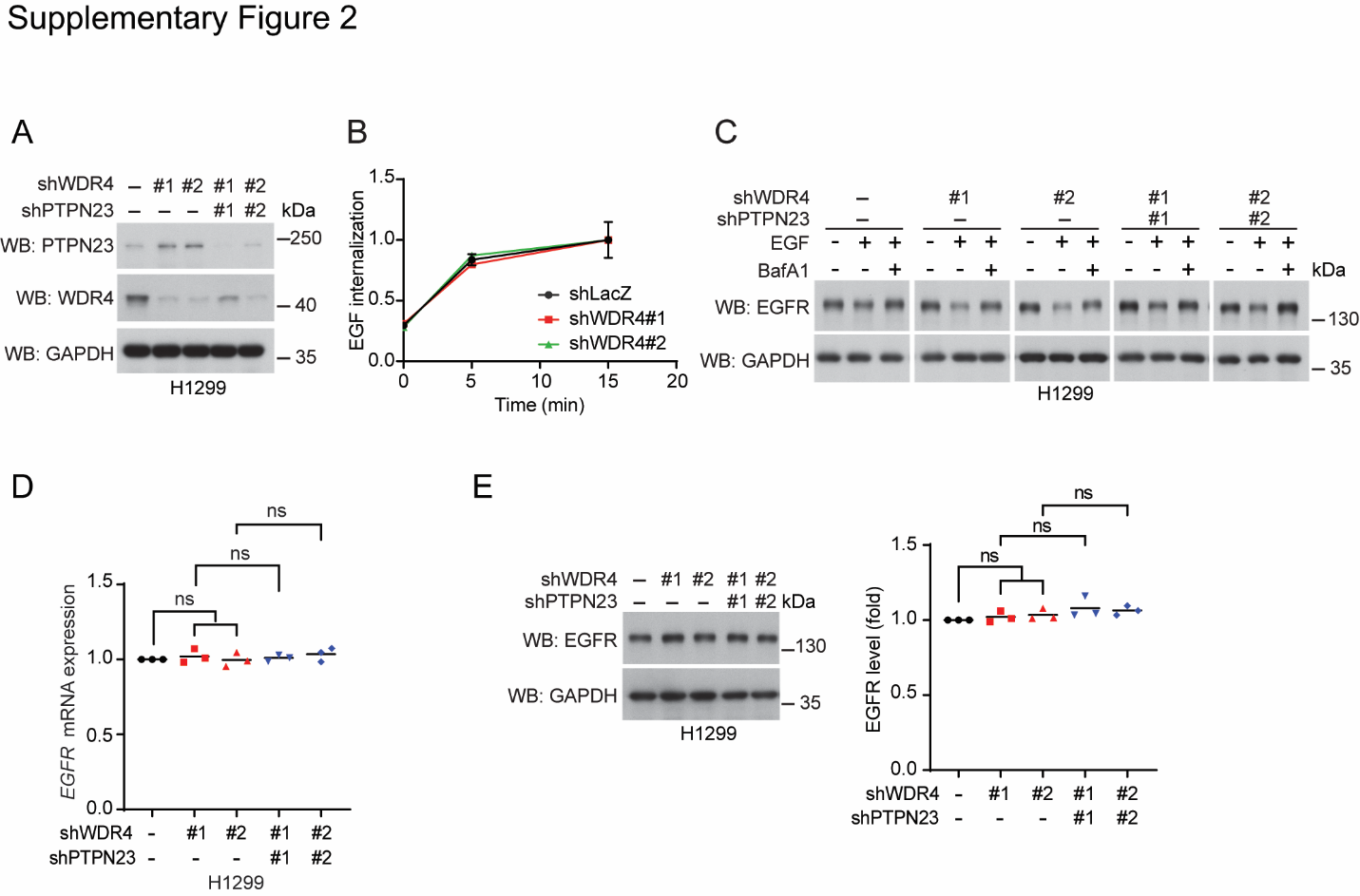
**

**Supplementary Figure S2. WDR4/PTPN23 axis diminishes EGF-induced EGFR lysosomal degradation without affecting EGF internalization.** (A) Western blot analysis of WDR4 and PTPN23 expression levels in H1299 cells stably expressing indicated shRNAs. (B) EGF internalization assay of indicated cells. The values of absorbance at 450 nm are plotted. Data are mean ± SD, n=3. (C) Western blot analysis of EGFR levels in H1299 cells stably expressing indicated shRNAs and treated with 100 ng/ml EGF and 100 nM bafilomycin A1 for 2 h. (D) *EGFR* mRNA levels in H1299 cells stably expressing indicated shRNAs. Data are represented as individual points and mean, n=3. P-values are determined by one-way ANOVA with Tukey’s post-hoc test. ns: not significant. (E) Western blot analysis of EGFR expression levels in H1299 cells stably expressing indicated shRNAs. The blots are representatives of three independent experiments and quantitative data are shown on the right. Data are represented as individual points and mean, n=3. P values are determined by one-way ANOVA with Tukey’s post-hoc test. ns: not significant.

**
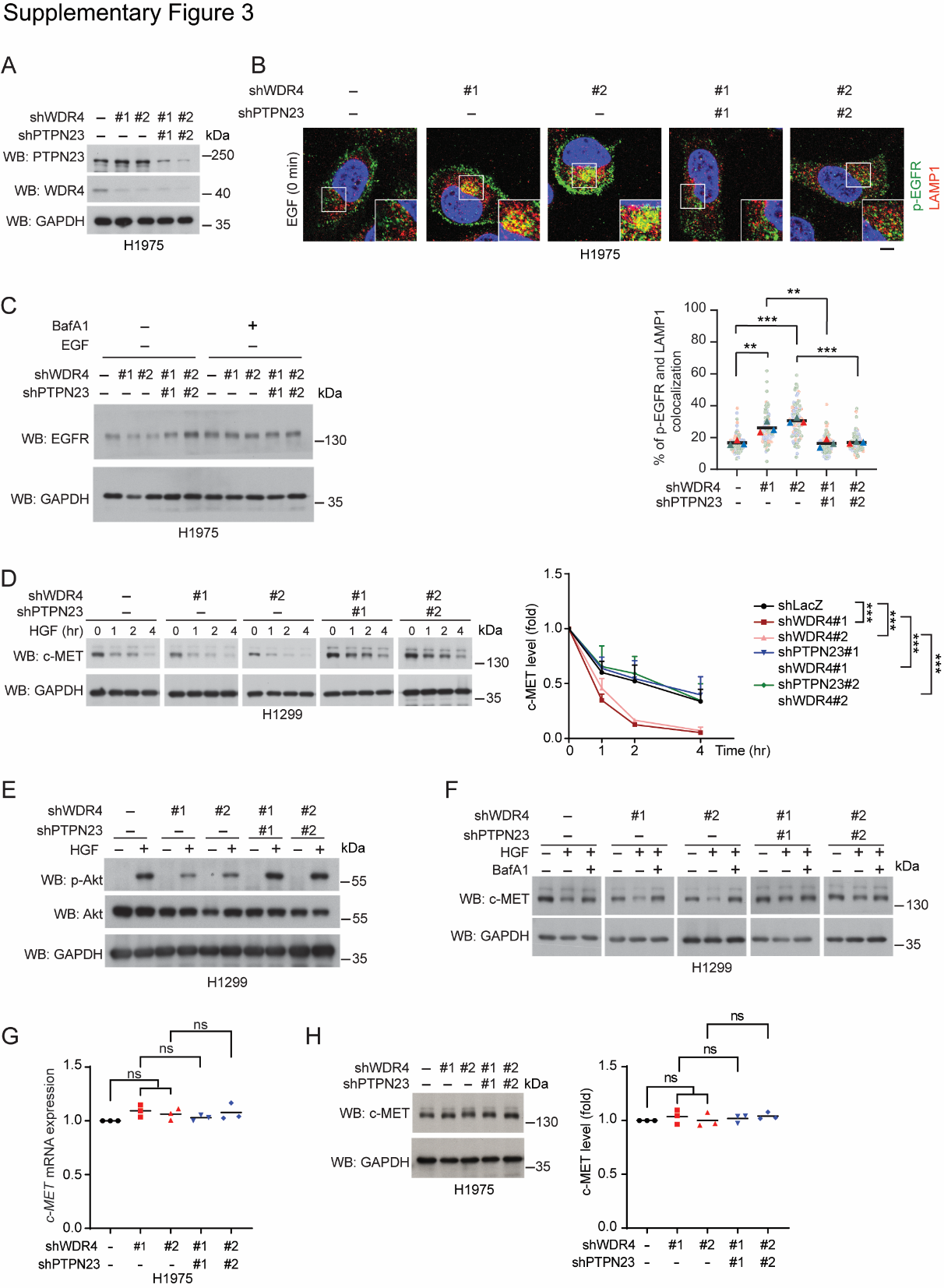
**

**Supplementary Figure S3. WDR4/PTPN23 axis diminishes lysosomal degradation of EGFR mutant and c-MET.** (A) Western blot analysis of WDR4 and PTPN23 expression levels in H1975 cells stably expressing indicated shRNAs. (B) Immunofluorescence staining for the colocalization of p-EGFR with LAMP1 in H1975 cells stably expressing indicated shRNAs without EGF treatment. Representative confocal images are shown on the top and quantitative data are on the bottom. Bar, 20 μm. Data are represented as mean, n=3 (30 cells per group per experiment were counted.). P values are determined by two-way ANOVA with Tukey’s post hoc test, **P<0.01, ***P<0.001. (C) Western blot analysis of EGFR levels in H1975 cells expressing indicated shRNAs without EGF treatment. (D) Western blot analysis of c-MET levels in H1299 cells expressing indicated shRNAs and treated with 100 ng/ml HGF for indicated time periods. Data are mean ± SD, n=3. P values are determined by two-way ANOVA with Tukey’s post hoc test, ***P<0.001. (E, F) Western blot analysis of indicated proteins in H1299 cells treated with 100 ng/ml HGF for 1 h (E), or 100 ng/ml HGF and 100 nM bafilomycin A1 for 3 h (F). (G) *c-MET* mRNA levels in H1975 cells stably expressing indicated shRNAs. Data are represented as individual points and mean, n=3. P-values are determined by one-way ANOVA with Tukey’s post-hoc test. ns: not significant. (H) Western blot analysis of c-MET expression levels in H1975 cells stably expressing indicated shRNAs. The blots are representatives of three independent experiments and quantitative data are shown on the right. Data are represented as individual points and mean, n=3. P values are determined by one-way ANOVA with Tukey’s post-hoc test. ns: not significant.

**
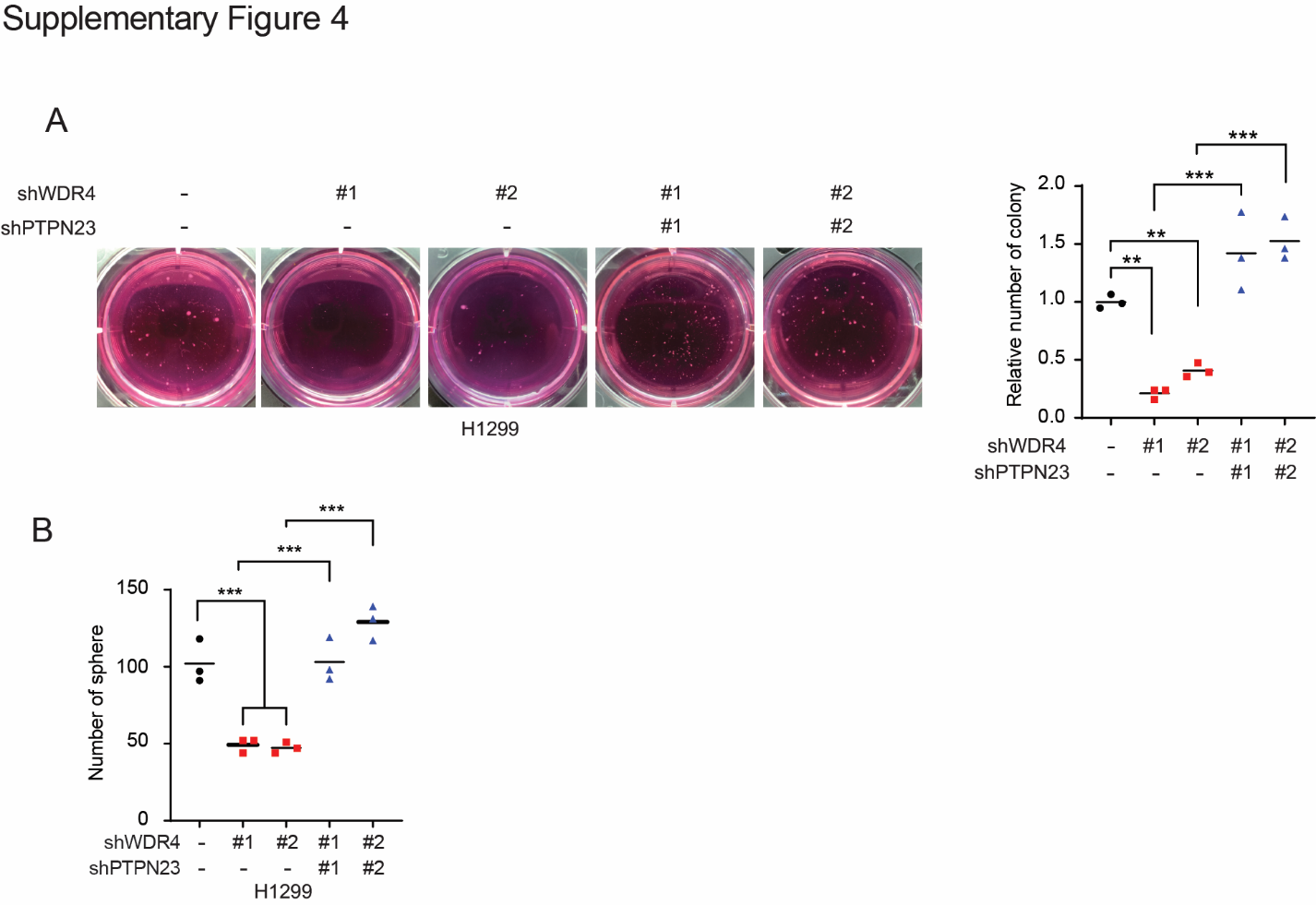
**

**Supplementary Figure S4. WDR4/PTPN23 axis promotes anchorage-independent growth and tumor sphere formation.** (A, B) Soft agar colony formation and tumor sphere formation abilities of H1299 cells stably expressing indicated shRNAs. Data are represented as individual points and mean, n=3. P values are determined by one-way ANOVA with Sidak’s multiple comparison test, **P<0.01, ***P<0.001.

**
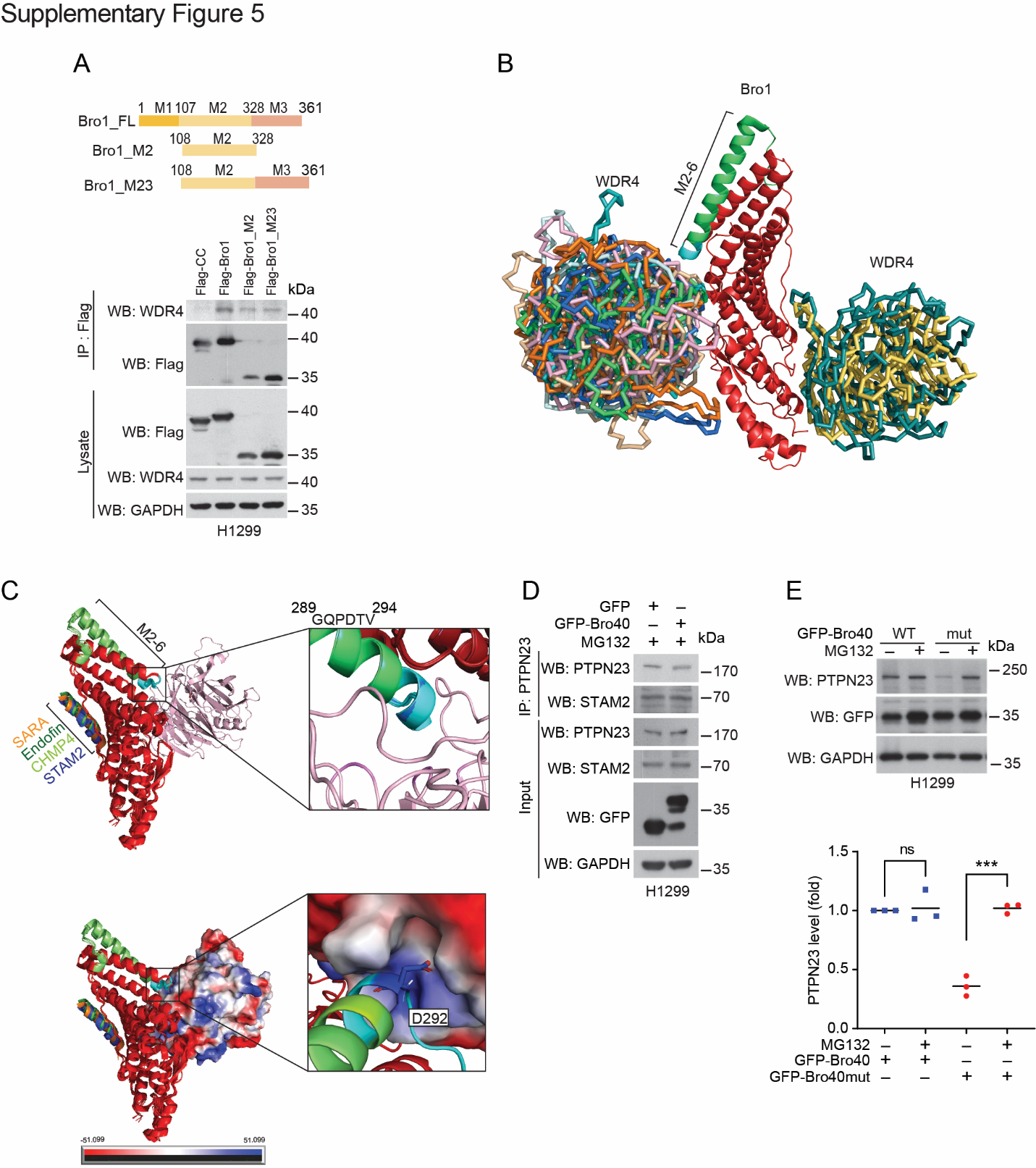
**

**Supplementary Figure S5. PTPN23 Bro40 binds WDR4 to block PTPN23 degradation.** (A) Immunoprecipitation analysis of the interaction between WDR4 and full length (FL) or truncated Bro1 domain in H1299 cells transfected with indicated constructs. The CC domain (residues 361-719) was used as a negative control. (B) In silico docked WDR4 and Bro1 domain complex. The 10 best WDR4-Bro1 docking models were used to demonstrate the favorable binding sides of Bro1. The 40-amino acid region is designated as M2-6 (green/cyan). (C) Top: Residues 289-294 (GQPDTV, cyan) of Bro1 are analyzed as the direct interacting region with WDR4 (lavender) in the WDR4-Bro1 ensemble. Bro1 and other ESCRT/endocytic protein structures were obtained from PDB and superimposed onto the predicted model. Bottom: The Aspartic acid at residue 292 is shown in stick. The blue painted region showed the positive charge carried by WDR4. (D) Immunoprecipitation analysis of the interaction between endogenous PTPN23 and endogenous STAM2 in H1299 cells transiently transfected with GFP-Bro40 or GFP-Bro40mut and treated with 5 μM MG132 for 16 h. (E) Western blot analysis of PTPN23 levels in H1299 cells transiently transfected with GFP-Bro40 or GFP-Bro40mut and treated with or without 5 μM MG132 for 16 h. The blots are representatives of three independent experiments and quantitative data are shown on the bottom. Data are represented as individual points and mean, n=3. P values are determined by one-way ANOVA with Tukey’s post-hoc test, ***P<0.001, ns: not significant.

**
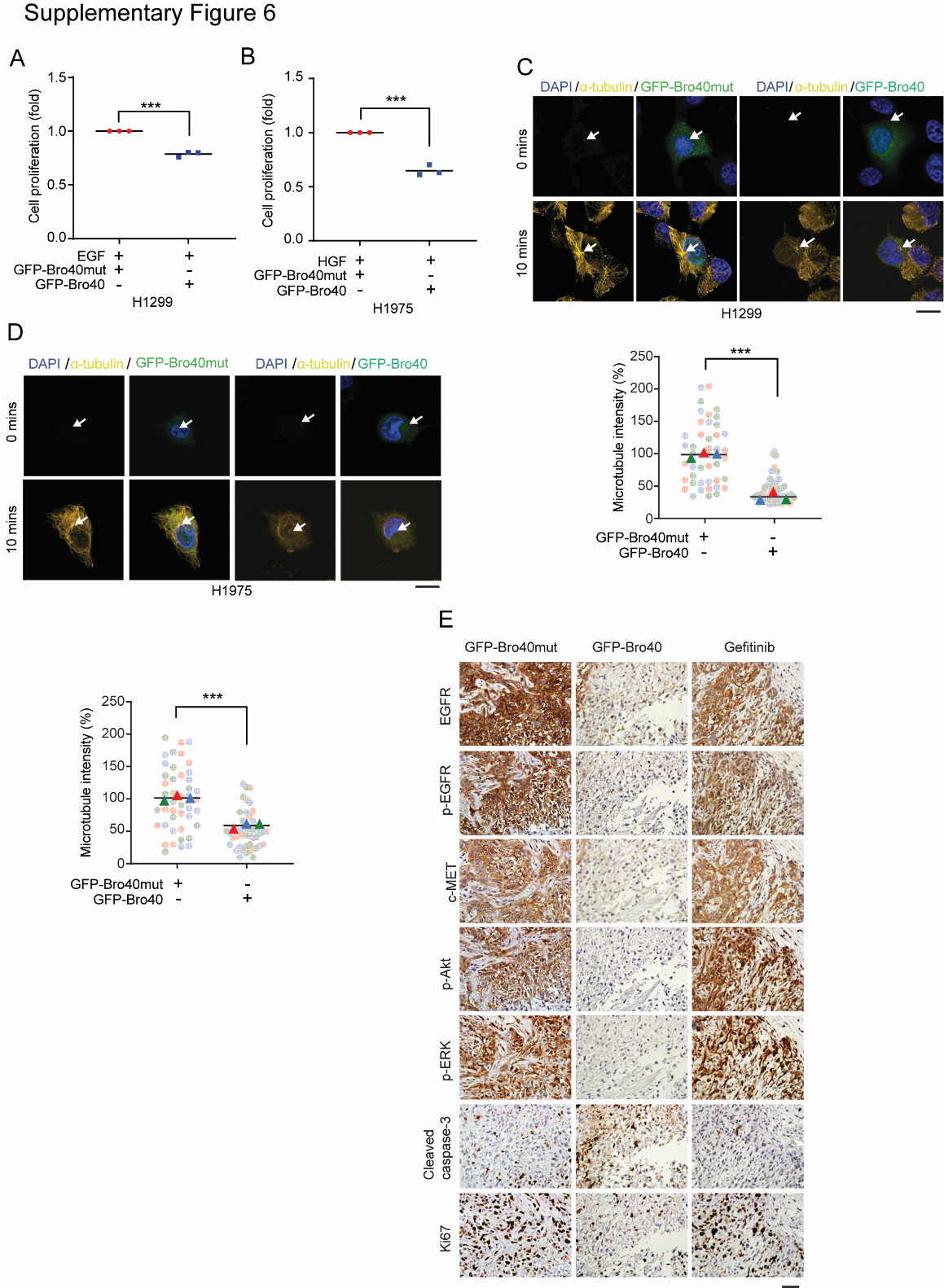
**

**Supplementary Figure S6. PTPN23 Bro40 downregulates EGFR and c-MET and elicits anti-tumor activities.** (A, B) Cell proliferation analysis of H1299 or H1975 cells transiently transfected with GFP-Bro40 or GFP-Bro40mut and treated with 100 ng/ml EGF or HGF for 24 h, respectively. Data are represented as individual points and mean, n=3. P values are determined by two-sided Student’s t-test, ***P<0.001. (C, D) Microtubule re-growth assay of H1299 or H1975 cells transiently transfected with GFP-Bro40 or GFP-Bro40mut. Representative images are on the top (C) or left (D). Arrows indicate the microtubule organizing center in transfected cells. Bar, 20 μm. Quantitative data for microtubule intensity at 10 min are shown on the bottom (C)or right (D). Data are represented as individual points and mean, n=3 (15 cells per group per experiment were counted). P values are determined by two-sided Student’s t-test, ***P<0.001. (E) Representative IHC images for the expression of indicated proteins in tumors derived from Fig. 7H. Bar, 20 μm.

**Supplementary Table S1: List of hits identified by ubiquitylome analyses**

Table S1-1: List of common proteins identified from WDR4 overexpression experiments

| Protein names | Ensemble  Protein ID | Ub Log2 Ratio (Normalized Protein level) | Protein Log2 Ratio |
| --- | --- | --- | --- |
| HIF1A | ENSG00000100644 | \| 7.198129486 \|  \| \| --- \| --- \| | -0.5542733 |
|  |  | \| 6.800676 \| \| --- \| | \| -0.15682011 \| \| --- \| |
| TNFRSF1A | ENSG00000067182 | 6.571373436 | -6.64385619 |
|  |  | 0.853159 | -0.552156356 |
| ANAPC5 | ENSG00000089053 | 7.310432456 | -6.64385619 |
|  |  | 6.16792 | -0.66657627 |
| AHR | ENSG00000106546 | \| 7.08020992 \|  \| \| --- \| --- \| | -0.43635373 |
|  |  | \| 5.970394 \|  \| \| --- \| --- \| | -6.64385619 |
| NCOA4 | ENSG00000266412 | \| 7.05313442 \|  \| \| --- \| --- \| | -0.40927823 |
|  |  | \| 6.651052 \| \| --- \| | \| -6.64385619 \| \| --- \| |
| CXADR | ENSG00000154639 | 7.037887831 | -0.39403164 |
|  |  | 1.259316 | -0.364013496 |
| SPRYD7 | ENSG00000123178 | 7.032211646 | -0.38835546 |
|  |  | 1.46433 | -0.015957574 |
| ALDH3B1 | ENSG00000006534 | \| 7.007869686 \| \| --- \| | \| -0.3640135 \| \| --- \| |
|  |  | \| 6.792657 \|  \| \| --- \| --- \| | -0.148800661 |
| ABCC3 | ENSG00000108846 | \| 6.993091631 \| \| --- \| | \| -0.34923544 \| \| --- \| |
|  |  | \| 6.891964 \| \| --- \| | -0.248107862 |
| GPR126; ADGRG6 | ENSG00000112414 | \| 6.98210659 \| \| --- \| | \| -0.3382504 \| \| --- \| |
|  |  | \| 9.183376 \| \| --- \| | \| -2.53951953 \| \| --- \| |
| METTL7B | ENSG00000170439 | \| 6.854752972 \| \| --- \| | \| -0.21089678 \| \| --- \| |
|  |  | \| 1.54122 \| \| --- \| | \| -1.531156057 \| \| --- \| |
| GALE | ENSG00000117308 | 6.873238543 | -0.22938235 |
|  |  | 6.978463 | -0.334607229 |
| SLC12A2 | ENSG00000064651 | \| 6.84145615 \|  \| \| --- \| --- \| | -0.19759996 |
|  |  | \| 1.480957 \| \| --- \| | \| -0.392137097 \| \| --- \| |
| KRT7 | ENSG00000135480 | \| 6.831563345 \| \| --- \| | \| -0.18770716 \| \| --- \| |
|  |  | \| 1.019407 \| \| --- \| | \| -0.115597447 \| \| --- \| |
| COX6B1 | ENSG0000012626 | \| 6.78150399 \| \| --- \| | \| -0.1376478 \| \| --- \| |
|  |  | \| 6.859771 \| \| --- \| | \| -0.215914857 \| \| --- \| |
| ITGB1 | ENSG00000150093 | \| 6.77675046 \| \| --- \| | \| -0.13289427 \| \| --- \| |
|  |  | \| 6.750106 \| \| --- \| | \| -0.106249498 \| \| --- \| |
| KIF11 | ENSG00000138160 | \| 6.736196362 \|  \| \| --- \| --- \| | -0.09234017 |
|  |  | \| 6.678903 \| \| --- \| | \| -0.035046947 \| \| --- \| |
| PTPN23 | ENSG00000076201 | \| 6.731589562 \| \| --- \| | \| -0.08773337 \| \| --- \| |
|  |  | \| 0.907219 \| \| --- \| | \| -0.77102743 \| \| --- \| |
| CD44 | ENSG00000026508 | \| 6.723944101 \| \| --- \| | \| -0.08008791 \| \| --- \| |
|  |  | \| 0.893308 \| \| --- \| | \| -0.005782353 \| \| --- \| |
| LAMA5 | ENSG00000130702 | \| 6.719376197 \| \| --- \| | \| -0.07552001 \| \| --- \| |
|  |  | \| 6.713308 \| \| --- \| | \| -0.069451881 \| \| --- \| |
| KRT8 | ENSG00000170421 | \| 6.714822711 \| \| --- \| | \| -0.07096652 \| \| --- \| |
|  |  | \| 6.699747 \| \| --- \| | \| -0.055891201 \| \| --- \| |
| KRT18 | ENSG0000011105 | \| 6.701247854 \| \| --- \| | \| -0.05739166 \| \| --- \| |
|  |  | \| 6.73159 \| \| --- \| | \| -0.087733372 \| \| --- \| |
| CTSB | ENSG00000164733 | \| 6.686312989 \| \| --- \| | \| -0.0424568 \| \| --- \| |
|  |  | \| 1.912831 \| \| --- \| | \| -0.118726939 \| \| --- \| |
| NRP1 | ENSG00000099250 | \| 6.656899227 \| \| --- \| | \| -0.01304304 \| \| --- \| |
|  |  | \| 1.512389 \| \| --- \| | \| -0.459972731 \| \| --- \| |
| NR0B1 | ENSG00000169297 | \| 2.31265342 \|  \| \| --- \| --- \| | -0.75389599 |
|  |  | \| 1.890376 \| \| --- \| | \| -2.023269779 \| \| --- \| |
| RAD18 | ENSG00000070950 | \| 2.22608605 \|  \| \| --- \| --- \| | -0.70604102 |
|  |  | \| 1.051603 \| \| --- \| | \| -0.209227962 \| \| --- \| |
| SCD | ENSG00000099194 | \| 2.081267382 \| \| --- \| | \| -1.72737955 \| \| --- \| |
|  |  | \| 0.811167 \| \| --- \| | \| -2.210896782 \| \| --- \| |
| VAMP2 | ENSG00000220205 | \| 1.79979491 \|  \| \| --- \| --- \| | -1.02326978 |
|  |  | \| 0.780219 \| \| --- \| | \| -0.049904906 \| \| --- \| |
| APLP2 | ENSG00000084234 | \| 1.284368548 \| \| --- \| | \| -1.48196851 \| \| --- \| |
|  |  | \| 2.058215 \| \| --- \| | \| -1.971430848 \| \| --- \| |
| CDC42SE2 | ENSG00000158985 | \| 1.066495412 \| \| --- \| | \| -0.91593574 \| \| --- \| |
|  |  | \| 0.700317 \| \| --- \| | \| -0.150400989 \| \| --- \| |
| ITGB4 | ENSG00000132470 | \| 1.008184236 \| \| --- \| | \| -0.3382504 \| \| --- \| |
|  |  | \| 1.228797 \| \| --- \| | \| -0.153606979 \| \| --- \| |
| SSBP1 | ENSG00000106028 | \| 0.895970187 \| \| --- \| | \| -0.00144342 \| \| --- \| |
|  |  | \| 0.983018 \| \| --- \| | \| -0.089267338 \| \| --- \| |
| PI4K2A | ENSG00000155252 | \| 0.843790796 \| \| --- \| | \| -0.1265805 \| \| --- \| |
|  |  | \| 0.672789 \| \| --- \| | \| -0.226003675 \| \| --- \| |
| EEA1 | ENSG00000102189 | \| 0.764580898 \| \| --- \| | \| -0.16812276 \| \| --- \| |
|  |  | \| 0.989529 \| \| --- \| | \| -0.046921047 \| \| --- \| |
| PCBP1 | ENSG00000169564 | \| 0.707421319 \| \| --- \| | \| -0.17136842 \| \| --- \| |
|  |  | \| 0.941918 \| \| --- \| | \| -0.112474729 \| \| --- \| |

Table S1-2: List of common proteins identified from WDR4 knockdown experiments

| Protein names | Protein ID | Ub Log2 Ratio (Normalized Protein level) | Protein Log2 Ratio |
| --- | --- | --- | --- |
| CYP51A1 | ENSG00000001630 | \| 6.74081792 \|  \| \| --- \| --- \| | -0.09696173 |
|  |  | \| 4.74398 \| \| --- \| | -2.052894948 |
| GJA1 | ENSG00000152661 | \| 7.232429944 \|  \| \| --- \| --- \| | -0.58857375 |
|  |  | \| 1.77207 \| \| --- \| | -1.569179503 |
| SLC30A1 | ENSG00000170385 | \| 0.894272493 \|  \| \| --- \| --- \| | -0.09850554 |
|  |  | \| 8.13391 \|  \| \| --- \| --- \| | -1.490050854 |
| RNF13 | ENSG00000082996 | \| 6.853084152 \|  \| \| --- \| --- \| | -0.20922796 |
|  |  | \| 8.0474 \| \| --- \| | -1.40354186 |
| HIF1A | ENSG00000100644 | \| 9.726997425 \|  \| \| --- \| --- \| | -3.08314124 |
|  |  | \| 7.66127 \| \| --- \| | -1.017417053 |
| SQSTM1 | ENSG00000161011 | \| 7.12986021 \|  \| \| --- \| --- \| | -0.48600402 |
|  |  | \| 7.4907 \| \| --- \| | -0.846843212 |
| VPS37C | ENSG00000167987 | \| 4.241236742 \|  \| \| --- \| --- \| | -3.45798964 |
|  |  | \| 7.48552 \| \| --- \| | -0.841662973 |
| UBE4B | ENSG00000130939 | \| 1.51393916 \|  \| \| --- \| --- \| | -0.81349944 |
|  |  | \| 7.42477 \| \| --- \| | -0.780908942 |
| PSMA6 | ENSG00000100902 | \| 0.823666788 \|  \| \| --- \| --- \| | -0.53324238 |
|  |  | \| 7.42229 \| \| --- \| | -0.778432211 |
| CTNNB1 | ENSG00000168036 | \| 7.055051623 \|  \| \| --- \| --- \| | -0.41119543 |
|  |  | \| 7.2587 \|  \| \| --- \| --- \| | -0.614845103 |
| PSMC3 | ENSG00000165916 | \| 2.210602799 \|  \| \| --- \| --- \| | -0.42275246 |
|  |  | \| 7.14203 \| \| --- \| | -0.498178735 |
| CD99L2 | ENSG00000102181 | \| 6.68334448 \| \| --- \| | -0.03948829 |
|  |  | \| 7.10581 \| \| --- \| | -0.461958547 |
| \| TXNL1 \| \| --- \| | ENSG00000091164 | \| 1.392033902 \|  \| \| --- \| --- \| | -0.45997273 |
|  |  | \| 7.07242 \| \| --- \| | -0.428565884 |
| HUWE1 | ENSG00000086758 | \| 6.949644582 \|  \| \| --- \| --- \| | -0.30578839 |
|  |  | \| 7.05889 \| \| --- \| | -0.415037499 |
| ITGB1 | ENSG00000150093 | \| 6.933683441 \|  \| \| --- \| --- \| | -0.28982725 |
|  |  | \| 1.28366 \|  \| \| --- \| --- \| | -0.388355457 |
| EIF3I | ENSG00000084623 | \| 7.032211646 \|  \| \| --- \| --- \| | -0.38835546 |
|  |  | \| 7.0228 \|  \| \| --- \| --- \| | -0.378944497 |
| MIB1 | ENSG00000101752 | \| 13.28771238 \|  \| \| --- \| --- \| | -6.64385619 |
|  |  | \| 7.00231 \|  \| \| --- \| --- \| | -0.358453971 |
| CPNE1 | ENSG00000214078 | \| 6.696751138 \|  \| \| --- \| --- \| | -0.05289495 |
|  |  | \| 0.76165 \| \| --- \| | -0.343732465 |
| USP5 | ENSG00000111667 | \| 0.818465637 \|  \| \| --- \| --- \| | -0.23446525 |
|  |  | \| 6.98028 \| \| --- \| | -0.336427665 |
| GNG12 | ENSG00000172380 | \| 1.152003093 \|  \| \| --- \| --- \| | -0.00144342 |
|  |  | \| 6.97665 \|  \| \| --- \| --- \| | -0.332789088 |
| ATP1B1 | ENSG00000143153 | \| 1.093859651 \|  \| \| --- \| --- \| | -0.35660555 |
|  |  | \| 0.95957 \|  \| \| --- \| --- \| | -0.329159664 |
| \| VIM \| \| --- \| | ENSG00000026025 | \| 0.927446973 \|  \| \| --- \| --- \| | -0.44614803 |
|  |  | \| 6.9568 \| \| --- \| | -0.312939312 |
| RAB10 | ENSG00000084733 | \| 0.793249155 \|  \| \| --- \| --- \| | -0.11247473 |
|  |  | \| 6.94786 \| \| --- \| | -0.304006187 |
| EPS15L1 | ENSG00000127527 | \| 0.753622938 \|  \| \| --- \| --- \| | -0.10469738 |
|  |  | \| 1.46379 \|  \| \| --- \| --- \| | -0.30222618 |
| HSP90AB1 | ENSG00000096384 | \| 1.428971305 \|  \| \| --- \| --- \| | -0.63486741 |
|  |  | \| 6.92665 \|  \| \| --- \| --- \| | -0.282789701 |
| ATP1A1 | ENSG00000163399 | \| 1.207466082 \|  \| \| --- \| --- \| | -0.31293931 |
|  |  | \| 1.02083 \| \| --- \| | -0.277533976 |
| FLNA | ENSG00000196924 | \| 0.757249866 \|  \| \| --- \| --- \| | -0.56064282 |
|  |  | \| 6.87663 \| \| --- \| | -0.232768963 |
| PTBP1 | ENSG00000011304 | \| 0.8622576 \|  \| \| --- \| --- \| | -0.14241704 |
|  |  | \| 6.86145 \|  \| \| --- \| --- \| | -0.217591435 |
| MPZL1 | ENSG00000197965 | \| 2.466542008 \|  \| \| --- \| --- \| | -1.44222233 |
|  |  | \| 6.85475 \|  \| \| --- \| --- \| | -0.210896782 |
| AMIGO2 | ENSG00000139211 | \| 6.883422315 \|  \| \| --- \| --- \| | -0.23956613 |
|  |  | \| 6.84643 \|  \| \| --- \| --- \| | -0.202571918 |
| RPN1 | ENSG00000163902 | \| 1.559646247 \|  \| \| --- \| --- \| | -0.27928376 |
|  |  | \| 6.8398 \|  \| \| --- \| --- \| | -0.195946441 |
| TSG101 | ENSG00000074319 | \| 1.392158754 \|  \| \| --- \| --- \| | -0.20756107 |
|  |  | \| 6.83485 \| \| --- \| | -0.190997225 |
| CDK9 | ENSG00000136807 | \| 6.775169424 \|  \| \| --- \| --- \| | -0.13131323 |
|  |  | \| 6.81848 \| \| --- \| | -0.174621396 |
| ENO1 | ENSG00000074800 | \| 1.206348881 \|  \| \| --- \| --- \| | -0.08467032 |
|  |  | \| 6.81522 \|  \| \| --- \| --- \| | -0.171368418 |
| BRAP | ENSG00000089234 | \| 0.697106574 \| \| --- \| | -0.32192809 |
|  |  | \| 6.80229 \|  \| \| --- \| --- \| | -0.158429363 |
| YWHAQ | ENSG00000134308 | \| 6.846428108 \|  \| \| --- \| --- \| | -0.20257192 |
|  |  | \| 6.79426 \|  \| \| --- \| --- \| | -0.150400989 |
| PRDX1 | ENSG00000117450 | \| 0.941306006 \|  \| \| --- \| --- \| | -0.14720211 |
|  |  | \| 6.78309 \| \| --- \| | -0.139235797 |
| ADRM1 | ENSG00000130706 | \| 6.962182048 \|  \| \| --- \| --- \| | -0.31832586 |
|  |  | \| 0.87732 \|  \| \| --- \| --- \| | -0.120294234 |
| PTRF; CAVIN1 | ENSG00000177469 | \| 6.768862551 \|  \| \| --- \| --- \| | -0.12500636 |
|  |  | \| 6.74855 \|  \| \| --- \| --- \| | -0.104697379 |
| SRP54 | ENSG00000100883 | \| 6.711795018 \|  \| \| --- \| --- \| | -0.06793883 |
|  |  | \| 6.74545 \|  \| \| --- \| --- \| | -0.10159814 |
| PTPN23 | ENSG00000076201 | \| 6.64819078 \| \| --- \| | -0.00433459 |
|  |  | \| 6.74236 \| \| --- \| | -0.098505545 |
| YWHAZ | ENSG00000164924 | \| 6.856423725 \|  \| \| --- \| --- \| | -0.21256754 |
|  |  | \| 0.87433 \|  \| \| --- \| --- \| | -0.09696173 |
| YWHAE | ENSG00000108953 | \| 6.821737915 \| \| --- \| | -0.17788173 |
|  |  | \| 0.67366 \| \| --- \| | -0.087733372 |
| DHX32 | ENSG00000089876 | \| 7.058893689 \|  \| \| --- \| --- \| | -0.4150375 |
|  |  | \| 6.71634 \| \| --- \| | -0.072482754 |
| ERGIC3 | ENSG00000125991 | \| 6.921390165 \|  \| \| --- \| --- \| | -0.27753398 |
|  |  | \| 6.70877 \| \| --- \| | -0.064917477 |
| RPL14 | ENSG00000188846 | \| 7.751659479 \|  \| \| --- \| --- \| | -1.10780329 |
|  |  | \| 6.70877 \| \| --- \| | -0.064917477 |
| RPLP0 | ENSG00000089157 | \| 1.378783599 \|  \| \| --- \| --- \| | -0.29335894 |
|  |  | \| 6.70275 \| \| --- \| | -0.058893689 |
| PRKDC | ENSG00000253729 | \| 6.713308071 \|  \| \| --- \| --- \| | -0.06945188 |
|  |  | \| 1.55513 \| \| --- \| | -0.057391664 |
| MYOF | ENSG00000138119 | \| 2.670435779 \|  \| \| --- \| --- \| | -0.30222618 |
|  |  | \| 1.27107 \| \| --- \| | -0.043943348 |
| RPL7A | ENSG00000148303 | \| 0.856241661 \|  \| \| --- \| --- \| | -0.7345631 |
|  |  | \| 6.68631 \|  \| \| --- \| --- \| | -0.042456799 |
| HSPA8 | ENSG00000109971 | \| 0.787706554 \|  \| \| --- \| --- \| | -0.20756107 |
|  |  | \| 6.65836 \| \| --- \| | -0.01449957 |
| SLC1A5 | ENSG00000105281 | \| 1.321928095 \|  \| \| --- \| --- \| | -0.09234017 |
|  |  | \| 6.65836 \| \| --- \| | -0.01449957 |
| HSP90B1 | ENSG00000166598 | \| 0.985729903 \|  \| \| --- \| --- \| | -0.3921371 |
|  |  | \| 2.23947 \| \| --- \| | -0.510457064 |
| DIP2B | ENSG00000066084 | \| 1.766585316 \|  \| \| --- \| --- \| | -0.63039393 |
|  |  | \| 2.15492 \|  \| \| --- \| --- \| | -0.785875195 |
| SLC1A4 | ENSG00000115902 | \| 1.24655736 \| \| --- \| | -1.00288828 |
|  |  | \| 1.0786 \| \| --- \| | -1.380821784 |
| HTRA2 | ENSG00000115317 | \| 0.942687347 \|  \| \| --- \| --- \| | -1.07400058 |
|  |  | \| 2.00915 \| \| --- \| | -0.582079992 |
| TUBB4B | ENSG00000188229 | \| 0.936075606 \|  \| \| --- \| --- \| | -0.32915966 |
|  |  | \| 1.98997 \|  \| \| --- \| --- \| | -0.092340172 |
| TUBB | ENSG00000196230 | \| 1.312029634 \|  \| \| --- \| --- \| | -0.51045706 |
|  |  | \| 1.71851 \|  \| \| --- \| --- \| | -0.289827252 |
| CRTAP | ENSG00000170275 | \| 6.081083929 \|  \| \| --- \| --- \| | -6.64385619 |
|  |  | \| 1.72476 \|  \| \| --- \| --- \| | -0.465938398 |
| RPS19 | ENSG00000105372 | \| 0.959132259 \|  \| \| --- \| --- \| | -0.13131323 |
|  |  | \| 1.51136 \|  \| \| --- \| --- \| | -0.051399153 |
| PHGDH | ENSG00000092621 | \| 6.978463419 \|  \| \| --- \| --- \| | -0.33460723 |
|  |  | \| 1.41293 \|  \| \| --- \| --- \| | -0.962969269 |
| FLRT3 | ENSG00000125848 | \| 6.84145615 \|  \| \| --- \| --- \| | -0.19759996 |
|  |  | \| 1.41065 \|  \| \| --- \| --- \| | -0.694321257 |
| PSMD11 | ENSG00000108671 | \| 6.985758984 \|  \| \| --- \| --- \| | -0.34190279 |
|  |  | \| 1.26138 \| \| --- \| | -0.325539348 |
| UCHL5 | ENSG00000116750 | \| 6.686312989 \|  \| \| --- \| --- \| | -0.0424568 |
|  |  | \| 1.12486 \|  \| \| --- \| --- \| | -0.318325858 |
| SOD1 | ENSG00000142168 | \| 6.765719423 \|  \| \| --- \| --- \| | -0.12186323 |
|  |  | \| 1.09433 \|  \| \| --- \| --- \| | -0.364013496 |
| FAT1 | ENSG00000083857 | \| 6.909200756 \|  \| \| --- \| --- \| | -0.26534457 |
|  |  | \| 1.09311 \|  \| \| --- \| --- \| | -0.081613766 |
| UBFD1 | ENSG00000103353 | \| 6.85141726 \|  \| \| --- \| --- \| | -0.20756107 |
|  |  | \| 1.0071 \|  \| \| --- \| --- \| | -0.496142467 |
| MPRIP | ENSG00000133030 | \| 0.703486034 \|  \| \| --- \| --- \| | -0.25670047 |
|  |  | \| 0.97346 \| \| --- \| | -0.058893689 |
| ABCC2 | ENSG00000023839 | \| 0.794151312 \|  \| \| --- \| --- \| | -0.44026348 |
|  |  | \| 0.93274 \| \| --- \| | -0.100051014 |
| FUNDC1 | ENSG00000069509 | \| 0.676566521 \|  \| \| --- \| --- \| | -0.25325728 |
|  |  | \| 0.92223 \| \| --- \| | -0.205896101 |
| PSMA7 | ENSG00000101182 | \| 7.269790472 \|  \| \| --- \| --- \| | -0.62593428 |
|  |  | \| 0.88462 \| \| --- \| | -0.634867407 |
| SSR4 | ENSG00000180879 | \| 0.796048477 \|  \| \| --- \| --- \| | -0.45008445 |
|  |  | \| 0.87501 \| \| --- \| | -0.467932448 |
| PSMA4 | ENSG00000041357 | \| 0.726669779 \|  \| \| --- \| --- \| | -0.56704059 |
|  |  | \| 0.81256 \| \| --- \| | -0.662003536 |
| CDC42 | ENSG00000070831 | \| 0.667220183 \|  \| \| --- \| --- \| | -0.35107444 |
|  |  | \| 0.80848 \|  \| \| --- \| --- \| | -0.131313235 |
| STX12 | ENSG00000117758 | \| 6.86648908 \|  \| \| --- \| --- \| | -0.22263289 |
|  |  | \| 0.79703 \|  \| \| --- \| --- \| | -0.512513651 |
| JAK1 | ENSG00000162434 | \| 1.415621941 \|  \| \| --- \| --- \| | -0.69665761 |
|  |  | \| 0.79592 \| \| --- \| | -0.17299399 |
| PSMA1 | ENSG00000129084 | \| 1.002359273 \|  \| \| --- \| --- \| | -0.71075571 |
|  |  | \| 0.78475 \|  \| \| --- \| --- \| | -0.76611194 |
| UFD1L; UFD1 | ENSG00000070010 | \| 7.162557248 \|  \| \| --- \| --- \| | -0.51870106 |
|  |  | \| 0.75587 \|  \| \| --- \| --- \| | -0.873027144 |
| PSMA5 | ENSG00000143106 | \| 4.050470359 \|  \| \| --- \| --- \| | -0.64611216 |
|  |  | \| 0.74217 \| \| --- \| | -1.005782353 |
| LBR | ENSG00000143815 | \| 2.152217127 \|  \| \| --- \| --- \| | -1.5691795 |
|  |  | \| 0.73978 \| \| --- \| | -0.118726939 |
| OPTN | ENSG00000123240 | \| 1.974871015 \|  \| \| --- \| --- \| | -1.58640592 |
|  |  | \| 0.73477 \| \| --- \| | -0.924125133 |
| BIRC6 | ENSG00000115760 | \| 0.727172124 \|  \| \| --- \| --- \| | -0.08008791 |
|  |  | \| 0.67047 \| \| --- \| | -0.10780329 |
| TSEN34 | ENSG00000170892 | \| 4.117806776 \|  \| \| --- \| --- \| | -3.07096652 |
|  |  | \| 0.70685 \| \| --- \| | -0.461958547 |
| AHNAK | ENSG00000124942 | \| 7.223778074 \|  \| \| --- \| --- \| | -0.57992188 |
|  |  | \| 0.67231 \| \| --- \| | -0.471928835 |
| LSS | ENSG00000160285 | \| 6.677425722 \| \| --- \| | -0.03356953 |
|  |  | \| 0.67151 \| \| --- \| | -0.375197235 |
| PSMC2 | ENSG00000161057 | \| 6.750105688 \|  \| \| --- \| --- \| | -0.1062495 |
|  |  | \| 0.66499 \| \| --- \| | 0.152003093 |

**Supplementary Table S2: Cox regression analysis of risk factors for cancer-related death in lung cancer patients**

| Characteristics | | Univariate analysis | | Multivariate analysis | |
| --- | --- | --- | --- | --- | --- |
|  |  | HR^A^ (95% Cl^B^) | P-value^C^ | HR^A^ (95% Cl^B^) | P-value |
| **WDR4 expression^D^** | WDR4^low^ | 1 | **<0.001** | 1 | **0.0353** |
|  | WDR4^high^ | 2.685  (1.551-4.904) |  | 1.908  (1.065-3.577) |  |
| **PTPN23**  **expression^D^** | PTPN23^low^ | 1 | **<0.001** | 1 | **<0.001** |
|  | PTPN23^high^ | 0.2261  (0.1130-0.4171) |  | 0.2738  (0.1329-0.5241) |  |
| **Age** | ≤ 65 | 1 | 0.6705 | -^F^ | -^F^ |
|  | > 65 | 1.111  (0.6846-1.816) |  | -^F^ |  |
| **Gender** | Female | 1 | **0.0083** | 1 | 0.3214 |
|  | Male | 1.942  (1.192-3.208) |  | 1.410  (0.7031-2.756) |  |
| **Smoker** | No | 1 | **0.0040** | 1 | 0.1548 |
|  | Yes | 2.083  (1.248-3.408) |  | 1.639  (0.8387-3.295) |  |
| **Type** | ADC | 1 | 0.6450 | -^F^ | -^F^ |
|  | SCC | 1.180  (0.5449-2.263) |  | -^F^ |  |
| **Stage** | Stage I-II | 1 | **0.0015** | 1 | 0.1218 |
|  | Stage III-IV | 2.214  (1.350-3.618) |  | 0.5055  (0.2165-1.237) |  |
| **T stage^E^** | Stage 1-2 | 1 | **0.0398** | 1 | 0.4354 |
|  | Stage 3-4 | 1.871  (0.9917-3.305) |  | 1.333  (0.6319-2.707) |  |
| **N stage^E^** | N0 | 1 | **<0.001** | 1 | **<0.001** |
|  | ≥ N1 | 3.029  (1.851-5.031) |  | 4.129  (1.776-8.979) |  |
| **M stage^E^** | M0 | 1 | **0.0011** | 1 | 0.1440 |
|  | ≥ M1 | 3.585  (1.545-7.334) |  | 1.908  (0.7599-4.396) |  |

A: HR: Hazard ratio. B: CI: Confidence interval. C: Bold values indicate statistical significance (p<0.05). D: WDR4 ^high^, high expression; WDR4 ^low^, low expression for WDR4. PTPN23 ^high^, high expression; PTPN23 ^low^, low expression for PTPN23. Expression was defined by IHC staining. E: T stage: primary tumor, N stage: lymph node metastasis; M stage: distant metastasis. F: The variables without significant HR in the univariate analysis were not included in the multivariate analysis.

**Supplementary Table S3: List of antibodies**

| Protein | Vendor | Catalogue Number | Species | Titer | Assay |
| --- | --- | --- | --- | --- | --- |
| EGF Receptor | Cell Signaling | 4267 | Rabbit | 1:1000 | WB |
|  |  |  |  | 1:50 | IHC |
| Phospho-EGF Receptor | Cell Signaling | 4407 | Rabbit | 1:1000 | WB |
|  | Cell Signaling | 4407 | Rabbit | 1:125 | IHC |
|  | GeneTex | GTX132810 | Rabbit | 1:100 | IF |
| Met | Cell Signaling | 8198 | Rabbit | 1:1000 | WB |
|  |  |  |  | 1:100 | IF |
|  |  |  |  | 1:150 | IHC |
| Akt | Cell Signaling | 9272 | Rabbit | 1:1000 | WB |
| Phospho-Akt | Cell Signaling | 4060 | Rabbit | 1:2000 | WB |
|  |  |  |  | 1:50 | IHC |
| GAPDH | GeneTex | GTX100118 | Rabbit | 1:10000 | WB |
| WDR4 | abcam | ab169526 | Rabbit | 1:10000 | WB |
|  |  | ab169526 |  | 1:250 | IHC |
|  |  | ab241297 |  |  | IP |
| PTPN23 | ProteinTech | 10472-I-AP | Rabbit | 1:1000 | WB |
|  |  |  |  | 1:100 | IHC |
|  |  |  |  |  | IP |
| p44/42 MAPK (Erk1/2) | Cell Signaling | 4695 | Rabbit | 1:1000 | WB |
| Phospho-Erk p44/42  MAPK (Erk1/2) | Cell Signaling | 4370 | Rabbit | 1:1000 | WB |
|  |  |  |  | 1:400 | IHC |
| Myc | ProteinTech | 16286-I-AP | Rabbit | 1:1000 | WB |
| 6X His | Takara | 631212 | Mouse | 1:5000 | WB |
| DYKDDDDK (Flag) Tag | Cell Signaling | 14793 | Rabbit | 1:5000 | WB |
| HA-Tag | Cell Signaling | 3724 | Rabbit | 1:5000 | WB |
| K48-linkage Specific Polyubiquitin | Cell Signaling | 8081 | Rabbit | 1:1000 | WB |
| IgG Isotype control | Cell Signaling | 3900 | Rabbit |  | IP |
| DDB1 | abcam | ab109027 | Rabbit | 1:50000 | WB |
| LAMP1 | abcam | 25630 | Mouse | 1:500 | IF |
| anti-Rabbit IgG HRP | GE Healthcare | NA934 | Donkey | 1:5000 | WB |
| anti-Mouse IgG HRP | GE Healthcare | NA931 | Sheep | 1:5000 | WB |
| anti-Rabbit IgG, Alexa Fluor 488 | Invitrogen | A11008 | Goat | 1:100 | IF |
| anti-Mouse IgG, Alexa Fluor 568 | Invitrogen | A11004 | Goat | 1:100 | IF |
| Ki-67 | Cell Signaling | 9449 | Mouse | 1:200 | IHC |
| Active Caspase-3 | Cell Signaling | 9664 | Rabbit | 1:2000 | IHC |
| GFP | Cell Signaling | 2956 | Rabbit | 1:1000 | WB |
|  | Santa Cruz | sc-9996 | Mouse | 1:500 |  |
| Veriblot (HRP for IP) | abcam | ab131366 |  | 1:5000 | WB |

**Supplementary Table S4: Sequences and sources of shRNAs**

| shRNA | Target sequence | SOURCE  (IDENTIFIER) |
| --- | --- | --- |
| PTPN23#1 | GACAACGACTTCATTTACCAT | National RNAi Core Facility, Academia Sinica, Taiwan |
| PTPN23#2 | GAGAACCCAGAAGCCTACAAT | National RNAi Core Facility, Academia Sinica, Taiwan |
| METTL1#1 | CCCACATTTCAAGCGGACAAA | National RNAi Core Facility, Academia Sinica, Taiwan |
| METTL1#2 | CGACTGGATGTGCACTCATTT | National RNAi Core Facility, Academia Sinica, Taiwan |

**Supplementary Table S5: Primer Sequences for qPCR**

| Gene name |  | Sequence (5’ to 3’) |
| --- | --- | --- |
| PTPN23 | F | TCCGTGTCCCACGAGACTTT |
|  | R | TTCATGCCCTCCTCAGACAC |
| GAPDH | F | GTCTCCTCTGACTTCAACAGCG |
|  | R | ACCACCCTGTTGCTGTAGCCAA |
| EGFR | F | AGGCACGAGTAACAAGCTCAC |
|  | R | ATGAGGACATAACCAGCCACC |
| c-MET | F | AGCGTCAACAGAGGGACCT |
|  | R | GCAGTGAACCTCCGACTGTATG |
| METTL1 | F | GGCTTCCAGAACATCGCCTGT |
|  | R | TGTCCGCTTGAAATGTGGGTCG |
| GAPDH | F | TGTTGCCATCAATGACCCCTT |
|  | R | CTCCACGACGTACTCAGCG |
