# Supplementary material for: PTPN23 ubiquitination by WDR4 suppresses EGFR and c-MET degradation to define a lung cancer therapeutic target
Source: Cell Death Dis. 2023 Oct 11;14(10):671. doi: 10.1038/s41419-023-06201-4 (PMC10567730; doi:10.1038/s41419-023-06201-4)

Original western blot

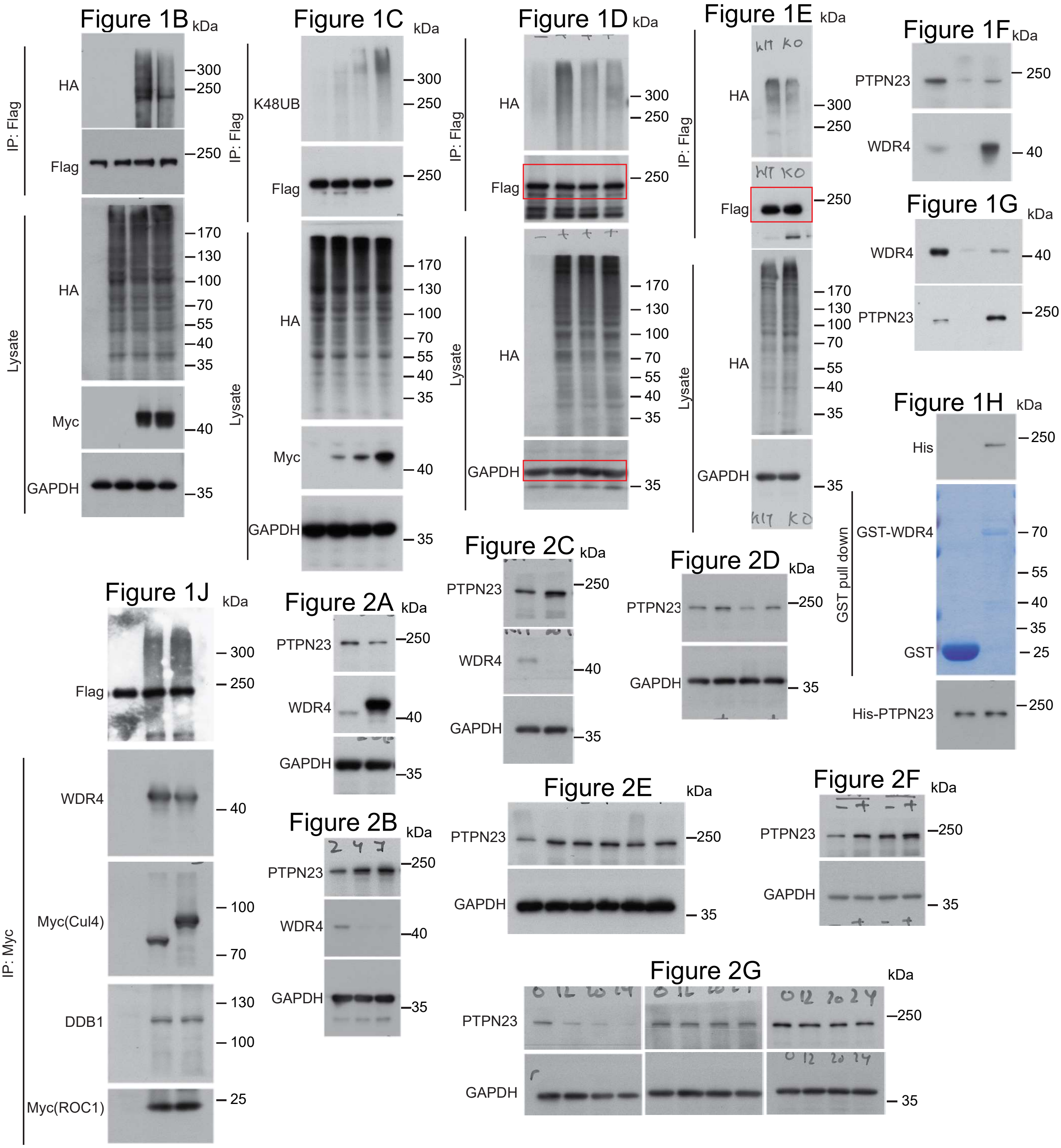

Figure 3C

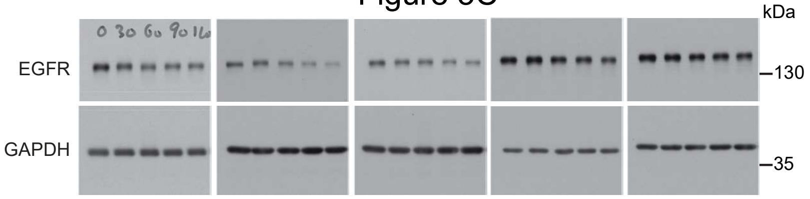

Figure 3D

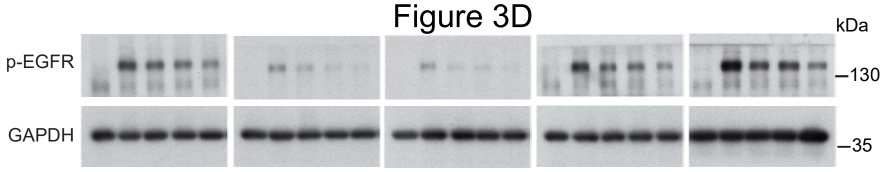

Figure 3E

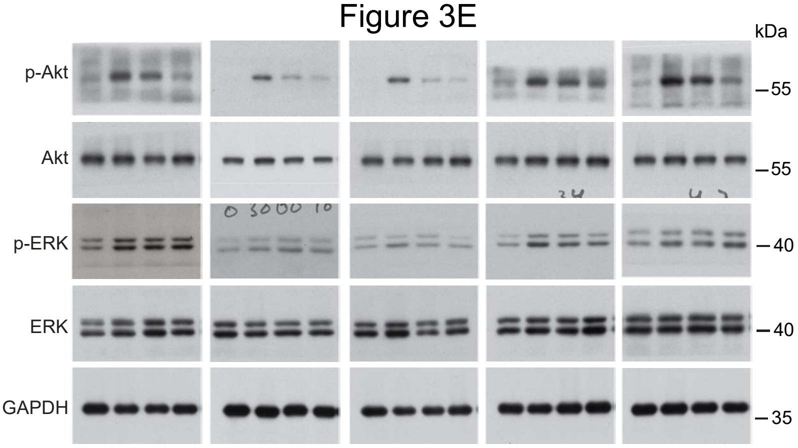

Figure 4B

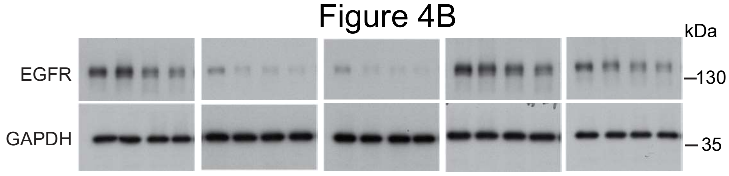

Figure 4C

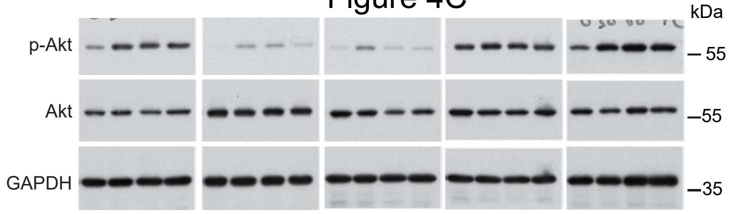

Figure 4D

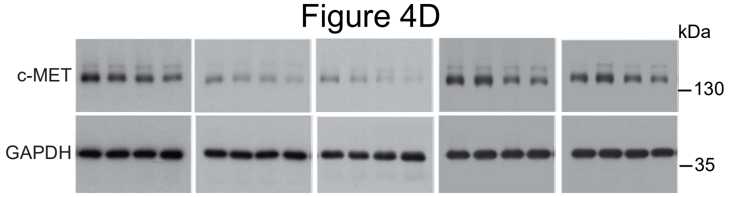

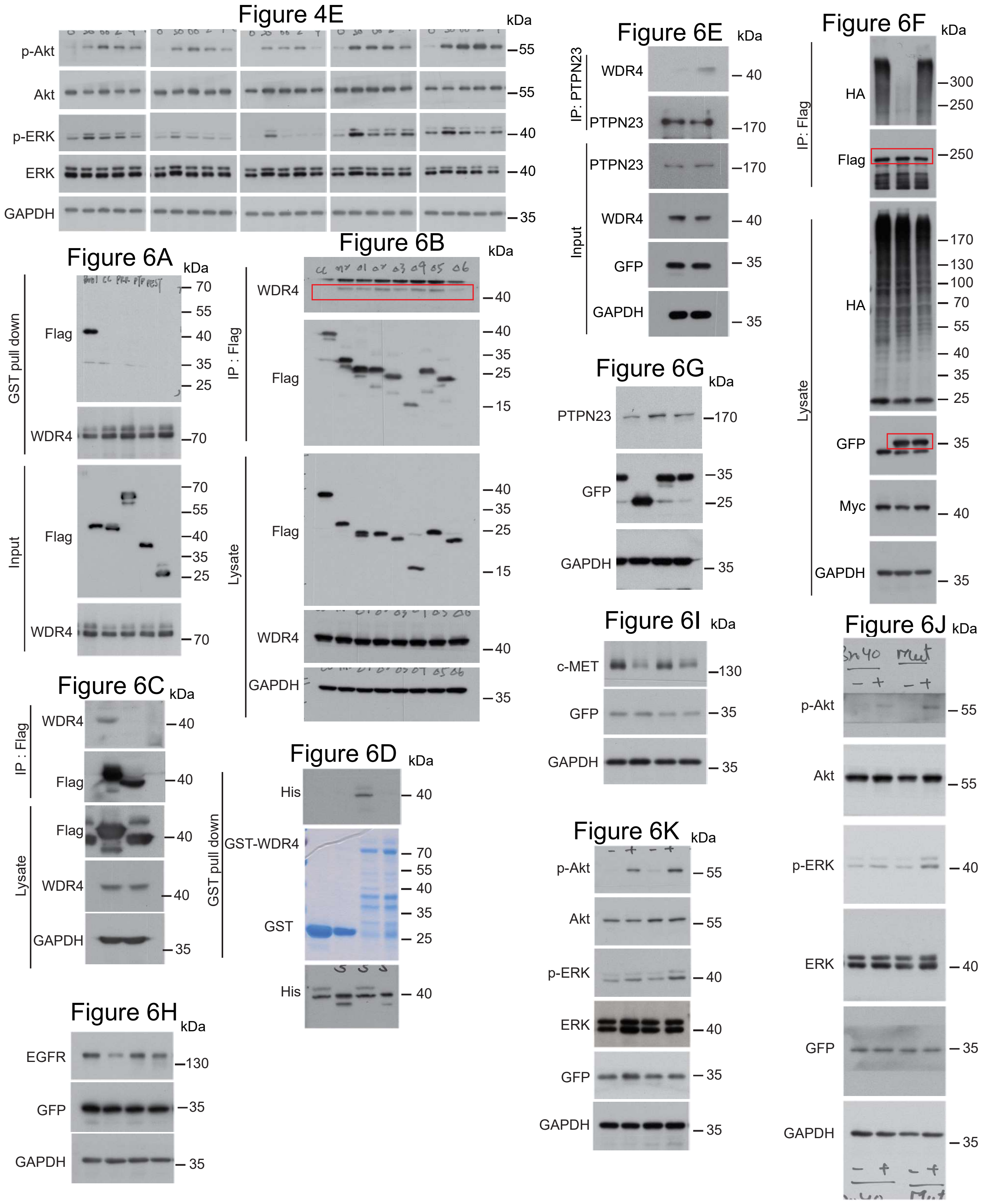
Figure 4E
kDa
p-Akt
Akt
p-ERK
ERK
GAPDH
55
55
40
40
35
Figure 6A
kDa
GST pull down
Flag
WDR4
Input
Flag
WDR4
70
55
40
35
25
70
70
55
40
35
25
70
Figure 6B
kDa
IP : Flag
WDR4
Flag
Lysate
Flag
WDR4
GAPDH
40
40
35
25
15
40
35
25
15
40
35
Figure 6C
kDa
IP : Flag
WDR4
Flag
Lysate
Flag
WDR4
GAPDH
40
40
40
40
35
Figure 6D
kDa
His
GST pull down
GST-WDR4
GST
His
40
70
55
40
35
25
40
Figure 6H
kDa
EGFR
GFP
GAPDH
130
35
35
Figure 6E
kDa
IP: PTPN23
WDR4
PTPN23
Input
PTPN23
WDR4
GFP
GAPDH
40
170
170
40
35
35
Figure 6G
kDa
PTPN23
GFP
GAPDH
170
35
25
35
Figure 6I
kDa
c-MET
GFP
GAPDH
130
35
35
Figure 6K
kDa
- + - +
p-Akt
Akt
p-ERK
ERK
GFP
GAPDH
55
55
40
40
35
35
Figure 6F
kDa
IP: Flag
HA
Flag
Lysate
HA
GFP
Myc
GAPDH
300
250
250
170
130
100
70
55
40
35
25
35
40
35
Figure 6J
kDa
Mut
- + - +
p-Akt
Akt
p-ERK
ERK
GFP
GAPDH
55
55
40
40
35
35
- + - +

Figure 6L

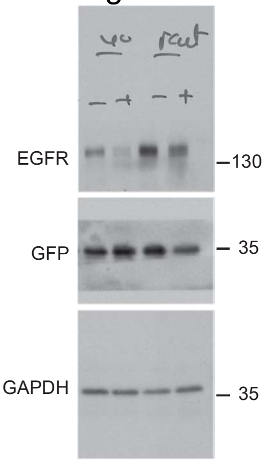

Figure 8A

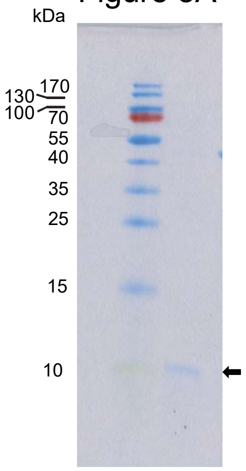

Figure 8B

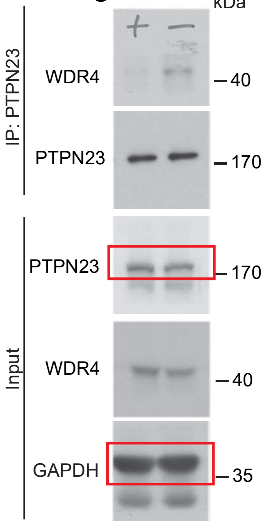

Figure 8C

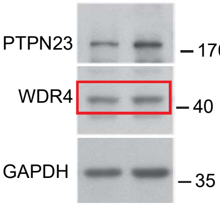

Figure S1B

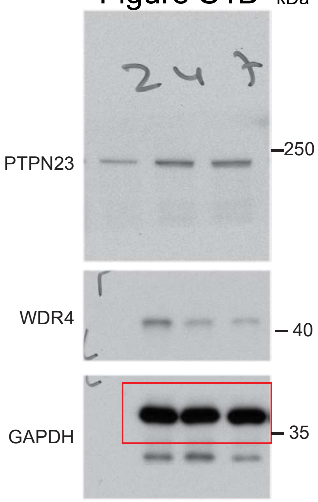

Figure S1C

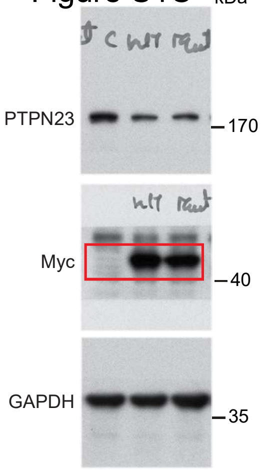

Figure S1D

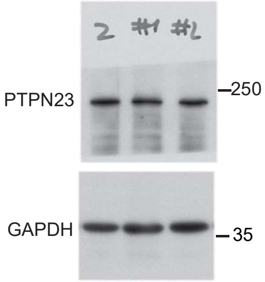

Figure S2C

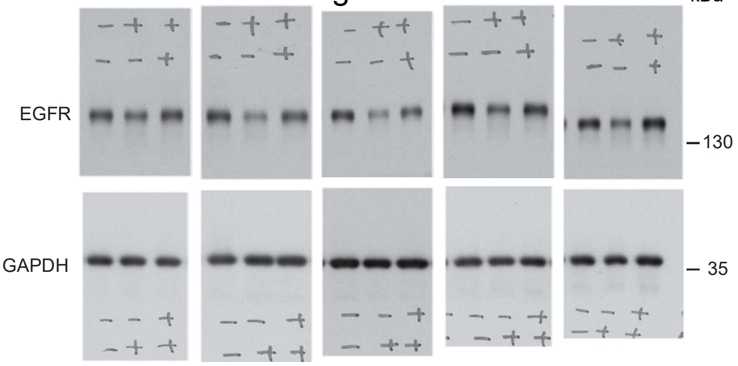

Figure S3A

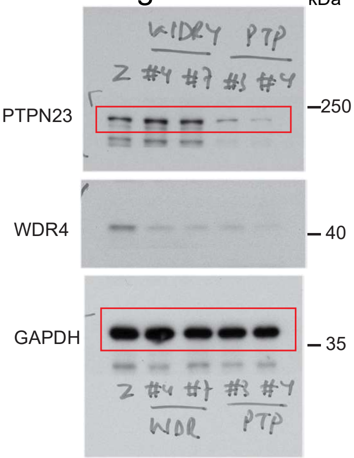

Figure S2A

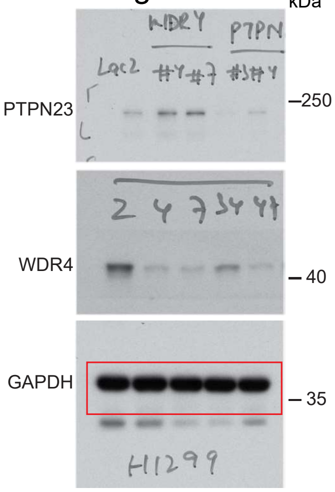

Figure S2E

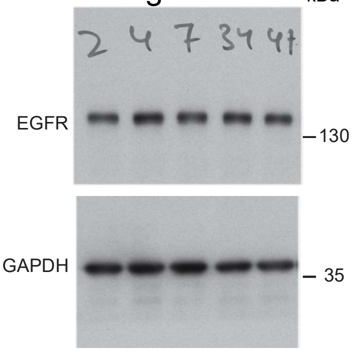

Figure S3C

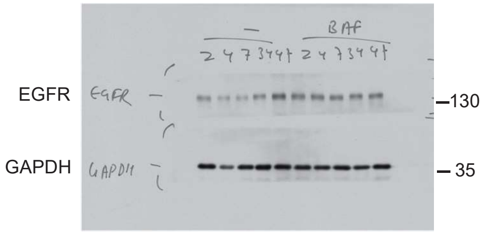

Figure S3D

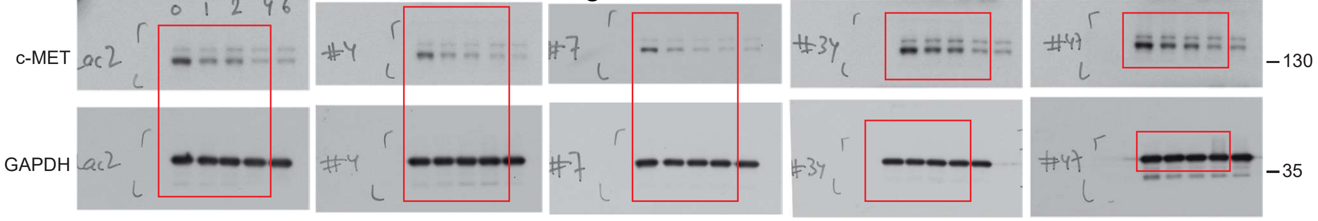

Figure S3E

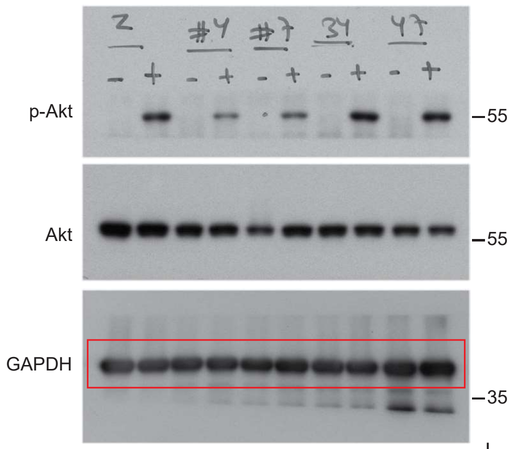

Figure S3F

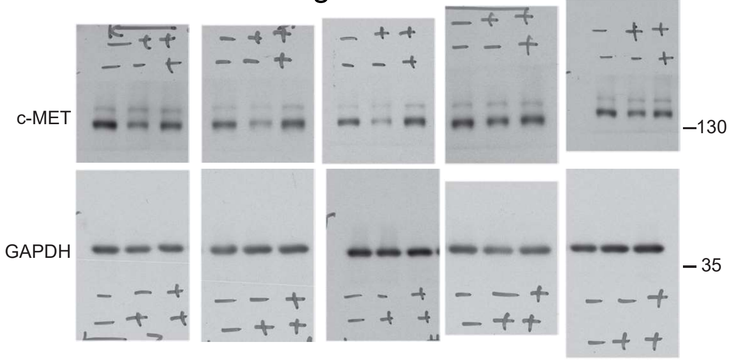

Figure S3H

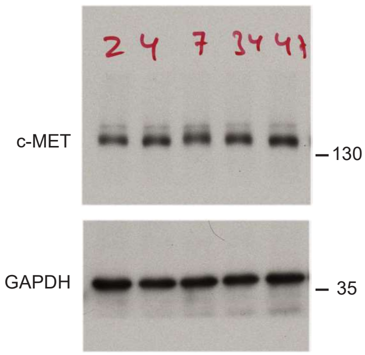

Figure S5A

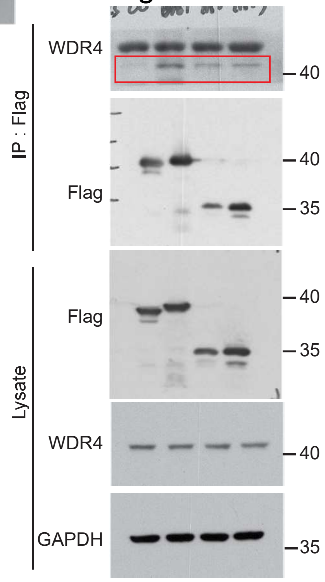

Figure S5D

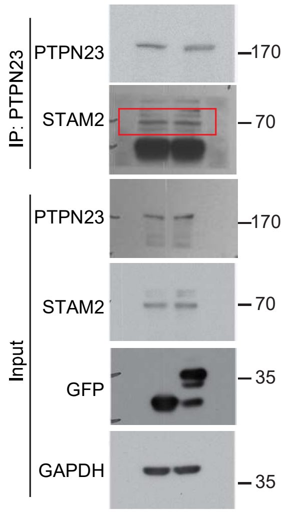

Figure S5E

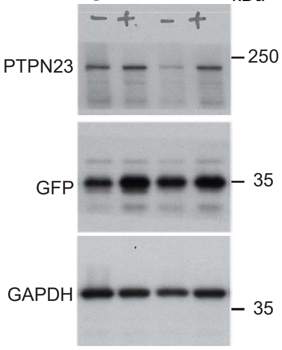

Supplement: Supplementary file 3 — original data file [file 41419_2023_6201_MOESM3_ESM.pdf]
